# Mathematical modelling with Bayesian inference to quantitatively characterize therapeutic cell behaviour in nerve tissue engineering

**DOI:** 10.1098/rsif.2023.0258

**Published:** 2023-09-06

**Authors:** Maxime Berg, Despoina Eleftheriadou, James B. Phillips, Rebecca J. Shipley

**Affiliations:** ^1^ Centre for Nerve Engineering, University College London, WC1E 6BT London, UK; ^2^ Department of Mechanical Engineering, University College London, WC1E 6BT London, UK; ^3^ School of Pharmacy, University College London, WC1N 1AX London, UK

**Keywords:** nerve tissue engineering, mathematical model, cell–solute model, Bayesian inference, model selection

## Abstract

Cellular engineered neural tissues have significant potential to improve peripheral nerve repair strategies. Traditional approaches depend on quantifying tissue behaviours using experiments in isolation, presenting a challenge for an overarching framework for tissue design. By comparison, mathematical cell–solute models benchmarked against experimental data enable computational experiments to be performed to test the role of biological/biophysical mechanisms, as well as to explore the impact of different design scenarios and thus accelerate the development of new treatment strategies. Such models generally consist of a set of continuous, coupled, partial differential equations relying on a number of parameters and functional forms. They necessitate dedicated *in vitro* experiments to be informed, which are seldom available and often involve small datasets with limited spatio-temporal resolution, generating uncertainties. We address this issue and propose a pipeline based on Bayesian inference enabling the derivation of experimentally informed cell–solute models describing therapeutic cell behaviour in nerve tissue engineering. We apply our pipeline to three relevant cell types and obtain models that can readily be used to simulate nerve repair scenarios and quantitatively compare therapeutic cells. Beyond parameter estimation, the proposed pipeline enables model selection as well as experiment utility quantification, aimed at improving both model formulation and experimental design.

## 1. Introduction

Peripheral nerve injury (PNI) repair is a fertile area of research [1,2]. PNIs are characterized by loss of sensory and motor functions and can cause chronic neuropathic pain. Three per cent of trauma results in PNIs [3], affecting the life of millions of people worldwide every year.

For less severe PNIs, natural nerve regeneration mechanisms are sufficient to induce functional repair; however, for more severe injuries, surgical intervention is often required. The autograft is the gold-standard treatment and involves transplanting a nerve section from a donor site into the nerve gap induced by the injury. The autograft therefore has numerous downsides which include the damages made to the donor site, the need for additional surgery and limited functional recovery [4].

Engineered tissues are being developed to address these issues and are considered a promising alternative to the autograft [1,5,6]. Cellular nerve conduits consist of a biomaterial tube which can either be hollow or filled with a variety of material components (e.g. hydrogels, fibres) to support regeneration [1]. These fillings can in turn be seeded with therapeutic cells that secrete an array of growth factors and provide trophic support for nerve regeneration. One example is engineered neural tissue (EngNT), which consists of aligned, cellular collagen hydrogel and has been developed at the UCL Centre for Nerve Engineering [5,7]. The aligned cells then produce, after transplantation and under the low-oxygen conditions of the injury site, a host of growth factors, including vascular endothelial growth factor (VEGF), which stimulate revascularization of the injury site.

A series of questions arise around the optimal design of a cellular nerve conduit including: what cell type, density and spatial distribution will lead to the best functional recovery? Answering such questions is challenging due to (i) our lack of fundamental understanding of the complex cascade of events that constitutes nerve regeneration and (ii) the large number of design parameters available. Tackling such questions using *in vivo* experiments in isolation is costly, time-consuming and requires a significant number of animal experiments.

**Figure 1.**
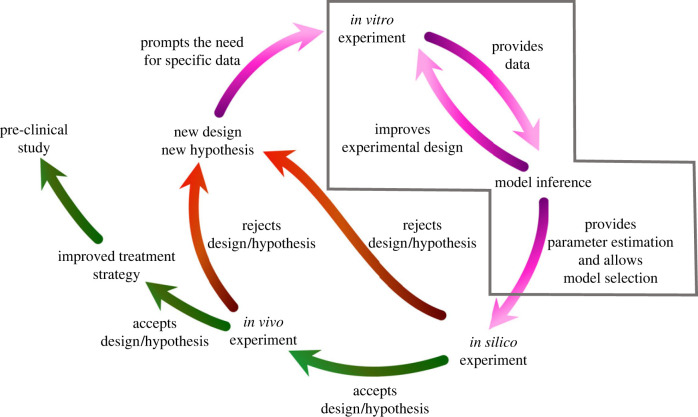
The combined experimental computational approach we propose to improve peripheral nerve injury treatment, with the focus of this work highlighted by the grey box.

Combining experiments with mathematical modelling provides an opportunity to address these challenges [8–10]. Mathematical models benchmarked against experimental data enable computational experiments to be performed to test the role of different biological and biophysical mechanisms, as well as to explore the impact of different design scenarios. A combined experimental-modelling framework allows for the efficient testing of hypotheses and design ideas, accelerating the translation of promising nerve repair solutions (figure 1).

One popular class of mathematical models used in tissue engineering are cell–solute models [8,11–13]. Such models consist of a set of continuous, coupled and generally nonlinear, partial differential equations describing the interplay between nutrients, the cell population(s) and the cell secretome in a given tissue. Cell–solute models describe a collection of interactions through a limited number of effective parameters. This makes them easy to implement and reasonably easy to interpret. One bottleneck of using such effective models, however, is that they rely heavily on the value of the parameters embedded within them, as well as the functional forms used to describe cell–solute interactions.

Parameter values and functional forms relating to cell metabolism and secretome are specific to an individual cell type in a particular environment and can be challenging to estimate. One option is to perform dedicated *in vitro* experiments in a highly controlled set-up, where inputs/outputs can be readily assessed. We have developed an *in vitro* set-up comprising multi-well plates where each well has a well-defined geometry and contains a thin layer of engineered tissue overlaid with a thick layer of culture media (figure 3*a*). Within this set-up, it is feasible to monitor cell activity under a range of culture conditions mimicking *in vivo* scenarios. A simultaneous benefit and limitation of such experiments is their limited spatio-temporal resolution—whereas this supports controlled and careful measurement, it remains a challenge to extrapolate these measured behaviours, generating uncertainties in mathematical model predictions which must be explored.

Bayesian inferences are a class of methods used to estimate parameter values and can take into account an arbitrary degree of uncertainty [14]. They have been used to build robust models in a wide range of fields [15–17], including tissue engineering [18,19], where it still remains uncommon due to the scarcity of combined modelling-experimental studies. The approach combines prior knowledge and new measurements to obtain posterior distributions of parameters rather than unique values, enabling the estimation of a range of statistics linked to each parameter (such as expected values or credible intervals). As well as providing quantitative information on specific parameter values, it also enables analysis of the relative contribution of different mechanisms and controllable parameters (such as operating parameters) in the model. This is highly valuable in informing the selection of functional forms to describe biomechanical mechanisms as well as design features. The trade-off, however, is that such analyses are stochastic by nature and may quickly become computationally expensive.

Building a mathematical model is therefore the result of the balance between the number of parameters in the model, the data availability to infer them and the computational cost of the associated simulations.

In this work, we focus on building a robust cell–solute model, relevant to therapeutic cells targeted in peripheral nerve tissue engineering, using Bayesian inferences integrated with cell outcome data from well-plate experiments *in vitro*, i.e. we focus on the highlighted section of the cycle displayed in figure 1. The outcome is a model that could be used to simulate cell–solute interactions in nerve repair scenarios, for instance in a nerve conduit.

We focus on collagen-based hydrogels and three cell types with high therapeutic potential—rat differentiated adipose-derived stem cells (dADSC, [8]), human neural stem cells (CTX, [13]) and rat Schwann cells (F7), though the framework we develop could be readily adapted to other cell and biomaterial combinations.

Besides parameter estimation we use our Bayesian approach to select the functional forms used in the cell–solute model, with a focus on the VEGF secretion rate. VEGF is an essential biochemical cue in blood vessel regeneration which is one of the first steps in nerve repair process. However, relatively few studies have sought to quantify the dependence of VEGF secretion rates on factors such as oxygen availability and cell density for individual cell types.

Finally, we quantify the impact of the operating/design parameters on cell behaviour. This will inform the design of future experiments to ensure they generate the most meaningful data on cell–solute interactions.

## 2. Material and methods

**Figure 2.**
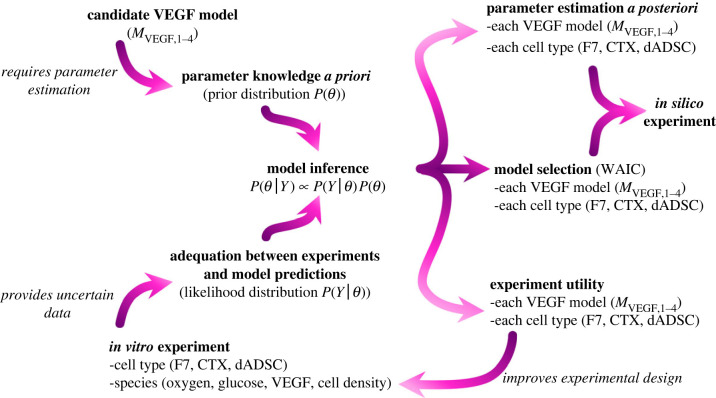
The framework developed in this work (highlighted by the grey box in figure 1) that integrates dedicated *in vitro* experiments with Bayesian inferences to build a mathematical model able to predict therapeutic cell behaviour.

This section presents the *in vitro* experiments (§2.1), the mathematical model derivation (§2.2) and the Bayesian inference methods (§2.3) that together form the basis of the framework we propose, which is detailed in figure 2.

### 2.1. *In vitro* experiments

**Figure 3.**
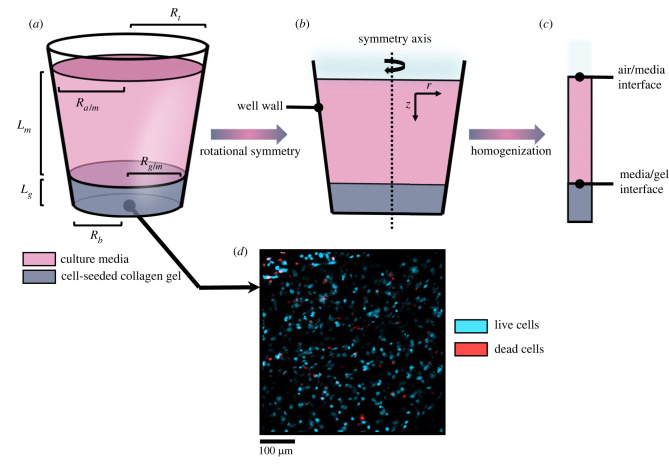
The well geometry (*a*) in three dimensions (3D), (*b*) after simplification via rotational symmetry in two dimensions (2D) and (*c*) after averaging to define an effective one-dimensional system (1D). (*a–c*) The culture media is depicted in pink and the cellular collagen gel is depicted in grey. (*d*) Confocal fluorescence microscopy image of F7 cell population in the gel after 24 h for an initial cell density of $31\;\mathrm{million}\;\mathrm{cells}\;ml^{-1}$. Living cells are shown in blue, dead cells are in red.

*In vitro* experiments were performed using standard 96-well plates. Each individual well had a truncated cone geometry (figure 3*a*) with fixed dimensions (table 1), with a layer of engineered tissue at the bottom (comprising of stabilized collagen-based cellular hydrogel, also referred to as the gel in this work, for thickness, see table 1) and a layer of culture media on top (figure 3*a*, for thickness, see table 1). Gels were fabricated using a range of seeded cell densities (table 2) and incubated at a range of ambient oxygen levels for 24 h (table 2). Oxygen concentration in the gel was monitored continuously for approximately 24 h (§2.1.2). Glucose and VEGF concentrations in the media were measured after 24 h (§2.1.3). Cell density in the gel was estimated after 24 h (§2.1.4). Sections 2.1.1 to 2.1.4 focus on F7 cell experiments; the counterpart data for dADSC and CTX cells have already been published [8,13].

**Table 1.** Well geometry dimension in millimetres (10^−3^ m).

cell type	top radius (*R*_*t*_)	bottom radius (*R*_*b*_)	gel thickness (*L*_*g*_)	media thickness (*L*_*m*_)	well height (*L*_*t*_)
F7	3.45	3.35	0.2	5.5	11.4
CTX	3.45	3.35	0.2	5.5	11.4
dADSC	4.03	3.31	0.18	3.82	12.4

**Table 2.** Experimental scenarios: ambient oxygen and seeded cell density tested for each cell type along with each species measured and the associated repeats. For each cell type, every combination of initial cell density and ambient oxygen level was tested.

cell type	ambient oxygen ($\%O_{2}$)	cell density (post-stabilization, 10^6^ cell ml^−1^)	species measured
F7 (rat Schwann cells)	1, 3, 7, 19	20, 31, 60	— oxygen gel concentration (*n* = 3)
			— glucose media concentration (*n* = 4)
			— VEGF media concentration (*n* = 4)
			— cell density (*n* = 4)
CTX (human neural stem cells)	1, 3, 7, 19	20, 31, 60	— oxygen gel concentration (*n* = 3)
			— glucose media concentration (*n* = 4)
			— VEGF media concentration (*n* = 4)
			— cell density (*n* = 4)
dADSC (rat differenciated adipose-derived stem cells)	1, 3, 5, 10, 16	39, 77, 154, 231, 385	— oxygen gel concentration (*n* = n.a.)
			— VEGF media concentration (*n* ∈ [3; 6])
			— cell density (*n* ∈ [3; 6])

#### 2.1.1. Engineered tissue

*Cell culture*. Rat Schwann cell line SCL4.1/F7 (Health Protection Agency) cells were grown in high glucose Dulbecco’s Modified Eagle’s Medium (DMEM) (Sigma Aldrich), supplemented with 10% (v/v) heat inactivated foetal bovine serum (FBS) (GibcoTM, ThermoFisher) and 1% (v/v) penicillin/streptomycin solution (GibcoTM, ThermoFisher), in tissue culture flasks (Greiner CELLSTAR, Sigma-Aldrich). Cells in flasks were maintained in a humidified incubator at 37°C and $5\%\;{\mathrm{CO}}_{2}$. The medium was replaced every 2–3 days until cells were approximately 80% confluent, as observed under phase-contrast microscopy. When passaging, cells were washed with phosphate-buffered saline (PBS) (GibcoTM, ThermoFisher) and trypsinized by the addition of 0.25% Trypsin-EDTA (GibcoTM, ThermoFisher) for 5 min at 37°C. Trypsin was inactivated by the addition of medium and the cell suspension was centrifuged at 300(*g*-force) for 5 min at room temperature.

This standard protocol was also used (with adaptations as appropriate) for dADSCs and CTXs [8,13].

*Fabrication of engineered tissue*. Stabilized cellular collagen gels were fabricated using protocols derived from [8,13,20]. Stabilized cellular collagen gels in *in vitro* wells were built to mimic the conditions in EngNT constructs. All gels were prepared using 80% v/v type I rat tail collagen (2 mg ml^−1^ in 0.6% acetic acid; First Link, UK) mixed with 10% v/v 10 × minimum essential medium (Sigma). The mixture was then neutralized using sodium hydroxide (NaOH; Sigma-Aldrich), and 10% v/v cell suspension was added to give a cell density of 0.5−1.5 × 10^6^ cells ml^−1^. Then 240 μl of this mixture was added per well in a 96-well plate. Gels were allowed to set at 37°C for 15 min. Stabilization was achieved using RAFT absorbers (Lonza Bioscience) for 15 min. The cell density post-stabilization was 20 −60 × 10^6^ cells ml^−1^.

#### 2.1.2. Ambient oxygen control and local oxygen measurement

A hypoxia workstation and incubator (HypoxyLab, Oxford Optronix, Oxford, UK) was used to control the ambient oxygen levels for the 24 h culture period (range $1\text{-}19\%O_{2}$). Local (or *in situ*) dissolved oxygen within the centre of the gels was measured using the integrated OxyLite (Oxford Optronix, UK) monitoring system. Fibre-optic oxygen probes were inserted into the centre of the three-dimensional stabilized collagen gels and the local oxygen concentration measured at 1 s time intervals (subsampled every half hour). The results were recorded on a standard laptop using Labview (National Instruments, Berkshire, UK).

#### 2.1.3. Glucose and VEGF measurement

*Glucose measurements*. Glucose consumption was quantified by an enzymatic assay (Glucose (HK) Assay Kit, GAHK20, Sigma Aldrich). Briefly, after 24 h, media samples were collected. The reconstituted reagent was added to each sample and the resulting solution was incubated for 15 min at room temperature. Optical absorbance was measured at 340 nm and was directly proportional to glucose concentration.

*VEGF measurements*. Secreted vascular endothelial growth factor-A (VEGF-A) concentrations after 24 h incubation of the cellular gels were determined by an enzyme-linked immunosorbent assay (ELISA). Media samples were collected, stored at $-20^{\circ }C$ and analysed with a VEGF-A sandwich ELISA kit (human and rat VEGF-A kits, RayBiotech, GA, USA).

#### 2.1.4. Cell density measurement

To assess cell viability, cultures were stained using Syto 21/Propidium Iodide (PI) (Sigma Aldrich) which allow for the simultaneous staining of viable and dead cells. The medium was removed from the gels, which were then washed three times with 200 μl of media ($37^{\circ }C$). (200 μl of Syto 21/PI solution for 15 min at $37^{\circ }C$). Gels were imaged using a confocal microscope (Zeiss-LSM710, Carl Zeiss, Germany) with 20× water immersion objectives.

### 2.2. Model derivation

#### 2.2.1. General equations

The well geometry is divided into two domains: the media *m* and the gel *g* (figure 3). Transport in both domains is assumed to be driven by diffusion (there is no imposed pressure gradient or flow) with diffusive fluxes modelled using Fick’s First Law. Both domains are treated as continua given the large cell densities (million cells ml^−1^) and we assume constant diffusion coefficients in each domain. Finally, we treat the problem as a superposition of independent binary solute/solvent mixtures, where each species is sufficiently dilute so that it does not change the medium density. Hence in the media we have

2.1 $$\partial_{t}C_{m}=D_{m}\nabla^{2}C_{m}-\lambda C_{m},$$

where *C*_*m*_ is the local solute (oxygen, glucose, VEGF) concentration in the media, *D*_*m*_ the associated diffusion coefficient and *λ* a linear degradation rate that represents the potential instability of the solute.

Similarly in the gel, we have

2.2 $$\partial_{t}C_{g}=D_{g}\nabla^{2}C_{g}-\lambda C_{g}+M(\{C_{g}\}),$$

where *C*_*g*_ is the local solute concentration or cell density field in the gel, *D*_*g*_ the associated diffusion coefficient. The term *M*({*C*_*g*_}) represents cell–solute interactions (e.g. consumption, secretion, proliferation, etc.), which are functions of {*C*_*g*_}, the set of all concentration/cell density fields in the gel. We note that in general *D*_*g*_ should depend on cell density; however, this dependence can be neglected for relatively low cell densities, which are the focus of this work [21].

Boundary conditions are imposed to mimic the physical and biological set-up. No-flux conditions are imposed on the well walls and base for all species so that

2.3 $$-D_{X}\nabla C_{X}\cdot n=0,$$

where *X* ∈ {*m*, *g*} and ***n*** is the outward pointing normal vector associated with the boundary. At the air/media interface, the nature of the boundary condition changes depending on the solute under consideration. For oxygen, we assume a fixed ambient oxygen concentration on the interface so that

2.4 $$C_{m}=C_{a/m},$$

where $C_{a/m}=\epsilon P_{a/m}$ is the concentration at the air/media interface, ϵ is the solubility factor associated with Henry’s Law in the media phase and *P*_*a*/*m*_ the partial pressure. For all other solutes, we assume a no-flux boundary condition which yields

2.5 $$-D_{m}\nabla C_{m}\cdot n=0.$$

Finally, at the gel/media interface, we assume zero mass flux for cells,

2.6 $$-D_{g}\nabla C_{g}\cdot n=0,$$

and mass flux continuity with no sorption for all solutes so that

2.7 $$-D_{m}\nabla C_{m}\cdot n=-D_{g}\nabla C_{g}\cdot n,$$

and to close the problem we assume that at equilibrium we have for all solutes

2.8 $$\lim_{t\to \infty }C_{m}=\eta_{g/m}C_{g},$$

where *η*_*g*/*m*_ corresponds to the chemical affinity (analogous to the partition coefficient). We note that the case *η*_*g*/*m*_ = 1 corresponds to continuity of the concentration fields between the gel and media.

#### 2.2.2. Effective equations

Equations (2.1)–(2.8) can be expressed in two dimensions given the axisymmetry of the geometry (figure 3*a*,*b*). It is possible to further simplify them to a one-dimensional description (figure 3*c*) assuming the well wall slope (*R*′ = −(*R*_*t*_ − *R*_*b*_)/*L*_*t*_) to be small and the gel layer thickness *L*_*g*_ to be small compared with the media layer thickness *L*_*m*_. Table 1 shows that these assumptions are reasonable for the cases considered in this work since *R*′ < 0.05 and *L*_*g*_/(*L*_*g*_ + *L*_*m*_) < 0.05. We apply asymptotic homogenization to equations (2.1)–(2.8) to obtain new, effective formulations (appendix A). For the media domain, this yields

2.9 $$\partial_{t}\langle C_{m}\rangle =2D_{m}\frac{R^{\prime }}{R}\partial_{z}\langle C_{m}\rangle +D_{m}\partial_{z}^{2}\langle C_{m}\rangle -\lambda \langle C_{m}\rangle ,$$

where 〈*C*_*m*_〉 represents the cross-section average solute concentration, *R* the local radius of the well and *z* the position along the axis of the well. The counterpart boundary conditions at the air/media interface become

2.10 $$\langle C_{m}\rangle =C_{a/m}$$

and

2.11 $$-D_{m}\partial_{z}\langle C_{m}\rangle =0,$$

for oxygen (equation (2.10)) and other solutes (equation (2.11)). For all solutes, at the gel/media interface, we now have an effective membrane condition

2.12 $$-D_{m}\partial_{z}\langle C_{m}\rangle =K\left(\langle C_{m}\rangle -\eta_{g/m}{\bar{C}}_{g}\right),$$

where ${\bar{C}}_{g}$ represents the average concentration in the gel layer and *K* = *D*_*g*_/*L*_*g*_ represents the effective permeability of the gel layer. Mass flux conservation is then enforced so that the integral balance in the gel layer becomes

2.13 $$\frac{d{\bar{C}}_{g}}{dt}=\frac{S_{g/m}K}{\Omega_{g}}(\langle C_{m}\rangle -\eta_{g/m}{\bar{C}}_{g})+M(\{{\bar{C}}_{g}\}),$$

where *S*_*g*/*m*_ is the cross-sectional area at the gel/media interface and $\Omega_{g}$ is the volume of the gel layer. For the case of cells, we consider *K* = 0.

Equations (2.9)–(2.13) form a nonlinear coupled set of effective diffusion–reaction equations that is solved numerically using a coarse-mesh finite volume approach (appendix B), for prescribed functional forms cell–solute interactions constitutive relationships, as described next.

#### 2.2.3. Functional forms for cell–solute constitutive relationships

Equations (2.9)–(2.13) describe the transport of a generic species in the gel and media. For each species of interest (here oxygen, glucose, VEGF, cells), the parameters in the model (such as diffusion coefficients) must be prescribed, as well as the functional form of $M(\{{\bar{C}}_{g}\})$ (denoted as *M*_ox_, *M*_glu_, *M*_VEGF_ and *M*_cell_, respectively) which encapsulates cell and solute processes as well as their interactions.

There are numerous approaches to describing these relationships in the literature [11,22–24]. We seek an approach which encapsulates the relevant biological mechanisms while minimizing the number of free parameters that must be prescribed. Where literature relationships are less well established and tested, we use a Bayesian approach (see §2.3) to select the form most relevant to our *in vitro* data.

Oxygen consumption is described using Michaelis–Menten kinetics, a simple and widely used expression in the literature for a variety of tissues [25–27] and given by

2.14 $$M_{\mathrm{ox}}=-{\bar{C}}_{\mathrm{cell}}M_{\mathrm{ox},\;\max}\frac{{\bar{C}}_{\mathrm{ox}}}{C_{\mathrm{ox},1/2}+{\bar{C}}_{\mathrm{ox}}},$$

where ${\bar{C}}_{\mathrm{cell}}$ and ${\bar{C}}_{\mathrm{ox}}$ are the average cell density and oxygen concentration in the gel, respectively. *M*_ox,max_ represents the maximum rate of oxygen consumption and *C*_ox,1/2_ the oxygen concentration for which the consumption rate is half its maximal value. The Michaelis–Menten kinetics is linear for oxygen concentration ${\bar{C}}_{\mathrm{ox}}\ll C_{\mathrm{ox},1/2}$ and constant for ${\bar{C}}_{\mathrm{ox}}\gg C_{\mathrm{ox},1/2}$ which represent the transition between a first-order kinetic and a saturated one.

Similarly, glucose consumption is modelled through Michaelis–Menten kinetics [28,29]. However, this time a term is added to account for consumption via both anaerobic respiration (under low-oxygen conditions) as well as respiration under oxygen-rich conditions

2.15 $$M_{\mathrm{glu}}=-{\bar{C}}_{\mathrm{cell}}M_{\mathrm{glu},\max}\frac{{\bar{C}}_{\mathrm{glu}}}{C_{\mathrm{glu},1/2}+{\bar{C}}_{\mathrm{glu}}}\left(1+A\frac{C_{\mathrm{ox},1/2}}{{\bar{C}}_{\mathrm{ox}}+C_{\mathrm{ox},1/2}}\right),$$

where ${\bar{C}}_{\mathrm{glu}}$ is the average glucose concentration in the gel. *M*_glu,max_ represents the maximum glucose consumption rate, *C*_glu,1/2_ the glucose concentration for which the consumption rate is half its maximal value and *A* is a factor which weights the contribution of anaerobic consumption (which is dependent on the local oxygen concentration) to the overall glucose consumption. The augmented glucose consumption due to the anaerobic mechanism is described as the difference between the baseline and oxygen-saturated scenarios (${\bar{C}}_{\mathrm{ox}}\to \infty$), i.e. $1-({\bar{C}}_{\mathrm{ox}}/({\bar{C}}_{\mathrm{ox}}+C_{\mathrm{ox},1/2}))=(C_{\mathrm{ox},1/2}/({\bar{C}}_{\mathrm{ox}}+C_{\mathrm{ox},1/2}))$.

Cells, on the other hand, can proliferate and die so that

2.16 $$M_{\mathrm{cell}}=P-Q,$$

where *P* and *Q* represent the proliferation and death rates, respectively. We assume cells proliferate through logistic growth, which is an established approach when modelling cell growth [30,31]

2.17 $$P=\gamma {\bar{C}}_{\mathrm{cell}}\left(\frac{{\bar{C}}_{\mathrm{ox}}}{{\bar{C}}_{\mathrm{ox},1/2}+{\bar{C}}_{\mathrm{ox}}}\right)\left(\frac{{\bar{C}}_{\mathrm{glu}}}{C_{\mathrm{glu},1/2}+{\bar{C}}_{\mathrm{glu}}}\right)\left(1-\frac{{\bar{C}}_{\mathrm{cell}}}{C_{\mathrm{cell},\max}}\right),$$

where *γ* represents the baseline proliferation rate and *C*_cell,max_ the threshold beyond which cells compete for space. The terms ${\bar{C}}_{\mathrm{ox}}/({\bar{C}}_{\mathrm{ox},1/2}+{\bar{C}}_{\mathrm{ox}})$ and ${\bar{C}}_{\mathrm{glu}}/(C_{\mathrm{glu},1/2}+{\bar{C}}_{\mathrm{glu}})$ relate high proliferation rates to high oxygen and glucose concentrations without adding new parameters into the model. To describe cell death, we follow the approach of Eleftheriadou *et al*. [13] and prescribe

2.18 $$Q={\bar{C}}_{\mathrm{cell}}\left(\delta_{0}+\delta_{\mathrm{ox}}\frac{C_{\mathrm{ox},1/2}}{{\bar{C}}_{\mathrm{ox}}+C_{\mathrm{ox},1/2}}+\delta_{\mathrm{glu}}\frac{C_{\mathrm{glu},1/2}}{{\bar{C}}_{\mathrm{glu}}+C_{\mathrm{glu},1/2}}\right),$$

where *δ*_0_ is the baseline death rate and where *δ*_ox_ and *δ*_glu_ are coefficients for cell death in low oxygen and glucose concentrations environment, respectively.

A number of functional relationships have been used to model VEGF secretion. Here four models have been selected (table 3). *M*_VEGF,1_ was selected for its simplicity (two parameters), *M*_VEGF,2_ because it is often encountered when modelling tissues [33] and *M*_VEGF,3_ and *M*_VEGF,4_ because they were derived specifically for nerve engineering purposes [8,13]. All four models take into account the upregulation of VEGF in low-oxygen conditions, via an upregulated secretion rate *β* and upregulation threshold *C*_ox,hypo_. The model *M*_VEGF,2_ introduces a baseline secretion rate *α* and a transition regime bounded by *C*_ox,hyper_ and *C*_ox,hypo_ between baseline and upregulated regimes. The transition between these two regimes is controlled by *ν*, with upregulated and baseline secretion rates related through the parameter *V* so that *β* = *α*(1 + *V*). By comparison, model *M*_VEGF,3_ introduces a dependence upon the initial cell density ${\bar{C}}_{\mathrm{cell},0}$ via parameters *α* and *V*. Finally, model *M*_VEGF,4_ introduces a dependency upon local cell density through a crowding threshold ${\bar{C}}_{\mathrm{cell},\mathrm{crowd}}$, i.e. a cell density beyond which VEGF secretion is hindered. We point out that parameter names and definitions have been adapted from their original publications in order to facilitate comparisons between models.

**Table 3.** Candidate VEGF secretion models used to represent therapeutic cell behaviour.

name	expression	mechanisms/characteristics	reference
*M*_VEGF,1_	$\begin{array}{cc} 0 & {\bar{C}}_{\mathrm{ox}}>C_{\mathrm{ox},\mathrm{hypo}}\\ \beta {\bar{C}}_{\mathrm{cell}}(1-({\bar{C}}_{\mathrm{ox}}/C_{\mathrm{ox},\mathrm{hypo}})) & {\bar{C}}_{\mathrm{ox}}\leq C_{\mathrm{ox},\mathrm{hypo}} \end{array}$	— low oxygen upregulation	[32]
		— linear upregulated state	
		— two parameters	
*M*_VEGF,2_	$\begin{array}{cc} \alpha {\bar{C}}_{\mathrm{cell}} & {\bar{C}}_{\mathrm{ox}}>C_{\mathrm{ox},\mathrm{hyper}}\\ \alpha {\bar{C}}_{\mathrm{cell}}(1+V{\left(\frac{C_{\mathrm{ox},\mathrm{hyper}}-{\bar{C}}_{\mathrm{ox}}}{C_{\mathrm{ox},\mathrm{hyper}}-C_{\mathrm{ox},\mathrm{hypo}}}\right)}^{\nu }) & C_{\mathrm{ox},\mathrm{hypo}}\leq {\bar{C}}_{\mathrm{ox}}\leq C_{\mathrm{ox},\mathrm{hyper}}\\ \alpha {\bar{C}}_{\mathrm{cell}}(1+V) & {\bar{C}}_{\mathrm{ox}}<C_{\mathrm{ox},\mathrm{hypo}} \end{array}$	— low oxygen upregulation	[33]
		— constant baseline state	
		— constant upregulated state	
		— controlled transition	
		— five parameters	
*M*_VEGF,3_	$\begin{array}{c} \alpha (C_{\mathrm{cell},0}){\bar{C}}_{\mathrm{cell}}(1+(1/2)V(C_{\mathrm{cell},0})(1+\tanh\nu (1-({\bar{C}}_{\mathrm{ox}}/C_{\mathrm{ox},\mathrm{hypo}}))))\\ \;\alpha (C_{\mathrm{cell},0})=\alpha_{0}+\alpha_{1}C_{\mathrm{cell},0}+\alpha_{2}C_{\mathrm{cell},0}^{2}\\ \;V(C_{\mathrm{cell},0})=V_{0}+V_{1}C_{\mathrm{cell},0} \end{array}$	— low oxygen upregulation	[8]
		— constant baseline state	
		— constant upregulated state	
		— controlled transition	
		— initial cell density effect	
		— seven parameters	
*M*_VEGF,4_	$\alpha ({\bar{C}}_{\mathrm{ox}}/C_{\mathrm{ox},\mathrm{hypo}}){\bar{C}}_{\mathrm{cell}}+\beta {\bar{C}}_{\mathrm{cell}}\;e^{-(({\bar{C}}_{\mathrm{ox}}/C_{\mathrm{ox},\mathrm{hypo}})+({\bar{C}}_{\mathrm{cell}}/{\bar{C}}_{\mathrm{cell},\mathrm{crowd}}))}$	— low oxygen upregulation	[13]
		— linear baseline state	
		— exponential upregulated state	
		— local cell density effect	
		— four parameters	

### 2.3. Bayesian inferences

The model (equations (2.9)–(2.18) and table 3) includes a number of unknown parameters that we seek to estimate based on the experimental measurements, while taking account of the underlying uncertainty in those measurements. To do so we employ Bayesian inferences. Based on Bayes’ theorem, we write

2.19 P(θ|Y)∝P(Y|θ)P(θ),

where ***Y*** is a vector of dimension *N*_*e*_ containing experimental measurements and where ***θ*** is a vector of dimension $N_{\theta }$ containing the parameters to be inferred. In this context, *P*(***θ***) corresponds to the prior distribution, i.e. the information known about the parameters before taking into account the new measurements (§2.3.1). Then, *P*(***Y***|***θ***) corresponds to the likelihood distribution, which serves as a proxy to represent the probability of a model prediction to accurately describe the measurements (§2.3.2). Finally, *P*(***θ***|***Y***) corresponds to the posterior distribution, which represents the knowledge of the parameters after taking into account the new measurements. To estimate parameters, we sample the posterior distribution multiple times using a Monte Carlo Markov chain (§2.3.3) and compute statistics based on the sample distribution, representative of the knowledge of the parameters *a posteriori* (§2.3.4) that can then be used as the basis for model selection (§2.3.5) and experimental design improvement §2.3.6).

#### 2.3.1. Prior distribution

Parameters to be inferred are placed into three categories:

(i) *Transport mechanisms* (table 7), which include diffusion coefficients in the media and gel (*D*_*m*_, *D*_*g*_), partition coefficients (*η*_*g*/*m*_) and degradation rates (*λ*) for each species.
(ii) *Cell–solute interactions* (table 8), which include all parameters involved in reaction terms (*M*_ox_, *M*_glu_, *M*_VEGF_ , *M*_cell_).
(iii) *Initial and boundary conditions* (table 9), which include the initial oxygen concentration (*C*_0,ox_), the initial glucose concentration (*C*_0,glu_), the initial cell density (*C*_0,cell_) and the oxygen concentration at the interface between air and media (*C*_*a*/*m*_).

We note that some additional parameters are fixed (e.g. well shape, media and gel thicknesses). Further, CTXs are conditionally immortalized so can only proliferate under certain conditions *in vitro*, and are inhibited from undergoing cell division after implantation into the body, hence we assume *γ* = 0. Similarly, oxygen and glucose do not degrade so that *λ* = 0 for both of them. Finally, we assume that there is no VEGF in the media at the outset (*C*_0,VEGF_ = 0).

In total, *N*_*θ*_ ≈ 30 parameters are inferred, this number changing slightly depending on the cell type and choice of VEGF secretion model.

For these parameters, we assume a positive, truncated normal distribution for simplicity, though we note that the approach could be readily adapted to any distribution. Only *α*_1_, *α*_2_ and *V*_1_ are associated with a normal distribution as we consider them as deviations of *α*_0_ and *V*_0_ in VEGF secretion model *M*_VEGF,3_ and therefore can take negative values.

We inform the mean and standard deviation for each parameter and initial and boundary condition using either values from the literature or by extrapolating them from the experimental data (appendix C).

Finally, all inferred parameters are assumed independent so that the joint prior distribution corresponds to the product of each individual distribution.

#### 2.3.2. Likelihood distribution

We describe the difference between model and experiment via a normally distributed noise with zero mean so that we have, for each data point of each species (oxygen, glucose, VEGF, cell density) and each cell type

2.20 $$Y_{k}-F_{k}(\theta )=N(0,\sigma_{e,k}^{2}),$$

where *Y*_*k*_ represents the experimental data point, *F*_*k*_(***θ***) represents the model prediction (i.e. the solution of equations (2.9)–(2.18) and table 3) and *σ*_e,*k*_ the standard deviation associated with data point *k*, which serves as a proxy for the confidence in the experiment. The likelihood distribution is then given by

2.21 $$P(Y_{k}|\theta )=N((Y_{k}-F_{k}(\theta )),\sigma_{e,k}^{2}).$$

We estimate *Y*_*k*_ and *σ*_e,*k*_, for each species (oxygen, glucose, VEGF, cell) and each cell type (F7, CTX, dADSC) using the mean and standard deviation associated with the experimental repeats (appendix D).

Similar to the prior distribution, we consider each data point as independent so that the joint likelihood distribution is the product of each individual distribution.

#### 2.3.3. Sampling posterior distribution

We sample the posterior distribution *P*(***θ***|***Y***), which we recall combines prior knowledge of the parameters and adequacy between experiment and model predictions (figure 2) using Monte Carlo Markov chains (MCMC, appendix E), for each VEGF model (table 3) and each cell type (table 2).

For each combination, we obtain a set of samples ${\left\{\theta_{i}\right\}}_{0<i\leq N_{s}}$ where *N*_*s*_ represents the number of times the posterior distribution was sampled. Each sample, ***θ***_*i*_ is a vector of dimension *N*_*θ*_ containing a single value for each of the parameters to be inferred, i.e. each parameter used to build the prior distribution (§2.3.1). Alternatively, ${\left\{\theta_{i}\right\}}_{0<i\leq N_{s}}$ can be interpreted as a *N*_*s*_ × *N*_*θ*_ matrix where each row corresponds to a single sample ***θ***_*i*_ and where each column contains all the sampled values for a specific parameter, that we label ***θ***_*j*_. In this context, *θ*_*ij*_ then represents the value of a specific parameter value within a given sample.

#### 2.3.4. Parameter estimation

For each parameter, we compute a set of statistics to evaluate the outcomes of the Bayesian inference process, for each cell type and each VEGF model. These are: the expected value of each parameter *a posteriori*

2.22 $$\mu_{\theta ,j}\approx \frac{1}{N_{s}}\sum_{i=1}^{i=N_{s}}\theta_{ij},$$

the marginal standard deviation *a posteriori*

2.23 $$\sigma_{\theta ,j}\approx {\left(\frac{1}{N_{s}}\sum_{i=1}^{N_{s}}{\left(\theta_{ij}-\mu_{\theta ,j}\right)}^{2}\right)}^{1/2},$$

and the 80% credible interval, i.e. the interval containing 80% of the posterior distribution defined as follows:

2.24 $${\left[\theta \right]}_{j}=[\theta_{\inf,j};\theta_{\sup,j}],$$

where

2.25 $$\theta_{\inf,j}\approx {\arg\;\min}_{\theta_{\inf}\in \theta_{j}}(\|ℵ(\{s\in \theta_{j},s<\theta_{\inf}\})-0.9N_{s}\|)$$

and

2.26 $$\theta_{\sup,j}\approx {\arg\;\min}_{\theta_{\sup}\in \theta_{j}}(\|ℵ(\{s\in \theta_{j},s>\theta_{\sup}\})-0.9N_{s}\|),$$

where ℵ represents the cardinal of the different sets.

#### 2.3.5. Vascular endothelial growth factor model selection and model averaging

We use the Watanabe–Akaike information criterion (WAIC, [34]) to evaluate the performance of each VEGF model, calculated for each cell type independently. WAIC takes into account both quality of fit and number of parameters and has the following expression:

2.27 $$\mathrm{WAIC}=-2\sum_{k=1}^{N_{\mathrm{VEGF}}}(\log(\bar{P(Y_{k}|\theta )})-\hat{\log(P(Y_{k}|\theta ))}),$$

where *N*_VEGF_ is the number of VEGF data points for each cell type, $\bar{P(Y_{k}|\theta )}=(1/N_{s})\sum_{i=1}^{N_{s}}P(Y_{k}|\theta_{i})$ and $\hat{\log(P(Y_{k}|\theta ))}=(1/N_{s})\sum_{i=1}^{N_{s}}(\log(P(Y_{k}{\left|\theta_{i})-\bar{\log(P(Y_{k}|\theta )}\right)}^{2}$ [35]. The smaller the WAIC value, the stronger the performance of the model so that we rank VEGF models accordingly.

The WAIC values associated with each model may be combined via an average posterior distribution [36]. This enables quantities such as expected values or marginal standard deviations for parameters shared by all the models (i.e. all parameters except the VEGF secretion-related parameters) to be calculated. This average posterior distribution has the form

2.28 $$\bar{P}(\theta |Y)=\sum_{l=1}^{l=4}P(\theta |Y,M_{\mathrm{VEGF},l})P(M_{\mathrm{VEGF},l}|Y),$$

where *P*(***θ***|***Y***, *M*_VEGF,*l*_) corresponds to the posterior distribution associated with each model *M*_VEGF,*l*_ (table 3), and *P*(*M*_VEGF,*l*_|***Y***) represents the posterior knowledge about the model itself. The latter may be calculated from Bayes theorem as

2.29 $$P(M_{\mathrm{VEGF},l}|Y)\propto P(Y|M_{\mathrm{VEGF},l})P(M_{\mathrm{VEGF},l}),$$

where *P*(***Y***|*M*_VEGF,*l*_) represents the accuracy of the model compared with data, and *P*(*M*_VEGF,*l*_) the *a priori* knowledge we have regarding the model itself.

We do not discriminate between the VEGF models presented in table 3 and assume them to be equally likely in representing VEGF secretion *a priori*. We use WAIC as a proxy to evaluate model performance and write

2.30 $$P(Y|M_{\mathrm{VEGF},l})P(M_{\mathrm{VEGF},l})\approx \frac{e^{-(1/2)\Delta {\mathrm{WAIC}}_{l}}}{\sum_{n=1}^{n=4}e^{-(1/2)\Delta {\mathrm{WAIC}}_{n}}},$$

where ΔWAIC_*l*_ represents the difference in the WAIC value between each *M*_VEGF,*l*_ and the best performing model. Using equations (2.29) and (2.30) in equation (2.28) allows us to estimate the average posterior distribution and to derive expected values, standard deviation and credible intervals by adapting equations (2.22)–(2.26) to the case of a sum of weighted distributions.

#### 2.3.6. Information gain and experiment utility

The relative entropy, defined as the Kullback–Leiber divergence associated with the posterior and prior distributions, is an established tool used to represent the utility of an experimental outcome in learning about model parameters [37–39]. It can be defined as

2.31 $$D_{\mathrm{KL}}(P(\theta |Y),P(\theta ))=-H(P(\theta |Y),P(Y|\theta )),$$

where *H*(*P*(***θ***|***Y***), *P*(***Y***|***θ***)) represents the cross entropy between likelihood and posterior distributions. Altogether, the Kullback–Leiber divergence can be interpreted as the information missing when using the prior distribution instead of the posterior distribution, and hence the information gain from performing the experiments. Following the definition of Shannon entropy, we have

2.32 $$H(P(\theta |Y),P(Y|\theta ))=\bar{\log(P(Y|\theta ))}-\log(P(Y)),$$

where $\bar{\log(P(Y|\theta ))}\approx \frac{1}{N_{s}}\sum_{i=1}^{N_{s}}\log(P(Y|\theta_{i}))$ and log(*P*(***Y***)) is the Bayesian model evidence [40], which is the normalizing constant of the posterior distribution associated with a given set of experiments, i.e. P(Y)=∫P(Y|θ)P(θ)dθ. We estimate this constant by adapting the MCMC used to sample the posterior distribution in §2.3.3 to the case of the prior distribution.

## 3. Results

### 3.1. Acellular case

We apply our approach to a preliminary case where no cells are seeded in the collagen gel. This allows us to estimate oxygen transport parameters for the first time in a simplified set-up. We then use such distributions as prior for the cellular case.

**Figure 4.**
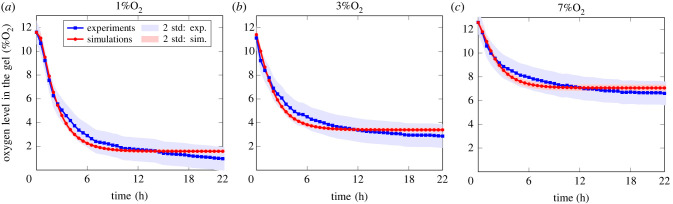
Comparison of oxygen concentration in the gel between experiment and simulation for the case of an acellular gel (*C*_0,cell_ = 0). (*a*) 1%O_2_, (*b*) 3%O_2_, (*c*) 7%O_2_. Number of repeats for oxygen measurements n.a. The red area corresponds to the standard deviation associated with the model predictions based on the posterior sample distribution, i.e. ${\left\{\theta_{i}\right\}}_{0<i\leq N_{s}}$. The blue area corresponds to the standard deviation associated with the experiments.

Figure 4 shows the measured oxygen levels in the gel over a 22 h period, maintained with ambient oxygen levels (or oxygen concentration at the air/media interface, i.e. *C*_*a*/*m*_) of 1%O_2_ (figure 4*a*), 3%O_2_ (figure 4*b*) and 7%O_2_ (figure 4*c*). Measured data are shown in blue and model predictions in red, based on expected values. Figure 4 shows good agreement between model and experiment for each ambient oxygen condition with a slight underestimation of the model around 6 h. The red area corresponds to the standard deviation associated with the model prediction based on the posterior sample distribution, i.e. ${\left\{\theta_{i}\right\}}_{0<i\leq N_{s}}$, and can be seen as a measure of the sensitivity of the predictions.

Table 4 shows the posterior expected value, marginal standard deviation and credible interval associated with the parameters, including initial and boundary conditions. We note that for the boundary condition, i.e. the oxygen concentration at the air/media interface, the expected value *a posteriori* remained close to its prior counterpart, which aligns with the posterior marginal standard deviation remaining close to its prior value. This is a direct effect of the higher confidence placed on the ambient oxygen level, which can be readily measured with high accuracy. We also see that the initial oxygen concentration, while remaining close to its prior value, has a drastically smaller standard deviation (this is also true of the associated partition coefficient). Indeed, the partition coefficient values inferred are close to one, indicating continuity between concentration fields between the gel and the media, and aligning with oxygen being a small molecule.

**Table 4.** Posterior estimates of oxygen transport parameters, and oxygen initial and boundary conditions in the acellular case. μ_*θ*_, *σ*_*θ*_ and [*θ*] correspond to the expected values, marginal standard deviation and credible intervals defined in equations (2.22), (2.23) and (2.24). Tables 7 and 9 recapitulate the parameter descriptions.

parameter	units	μ_*θ*_	*σ*_*θ*_	[*θ*]
*D*_*m*,ox_	10^−9^ m^2^ s^−1^	1.99	0.05	[1.91, 2.05]
*D*_*g*,ox_	10^−10^ m^2^ s^−1^	4.51	0.57	[3.87, 5.39]
*η*_*g*/*m*,ox_	n.a.	0.96	0.02	[0.94, 0.98]
*C*_0,ox_	4.21 × 10^−4^ kg m^−3^	11.54	0.30	[11.15, 12.85]
*C*_*a*/*m*_	4.21 × 10^−4^ kg m^−3^	1.31/3.16/6.87	0.06/0.07/0.09	[1.23/3.07/6.76, 1.40/3.24/6.98]

Next, the expected values and marginal standard deviations reported in table 4 for the diffusion coefficient in the media, gel and partition coefficient are used as prior distribution for the cellular gel cases.

### 3.2. Cellular case

We use the samples obtained from the posterior distributions (§2.3.3) to estimate, for each cell type (F7, CTX, dADSC) and for each VEGF secretion model (table 3), the posterior expected values (equation (2.22)), the posterior marginal standard deviations (equation (2.23)) and the credible intervals (equation (2.24)) of all the parameters in the model (§2.3.1). Such estimates are reported in electronic supplementary material (tables S1–S12).

#### 3.2.1. Model selection

**Figure 5.**
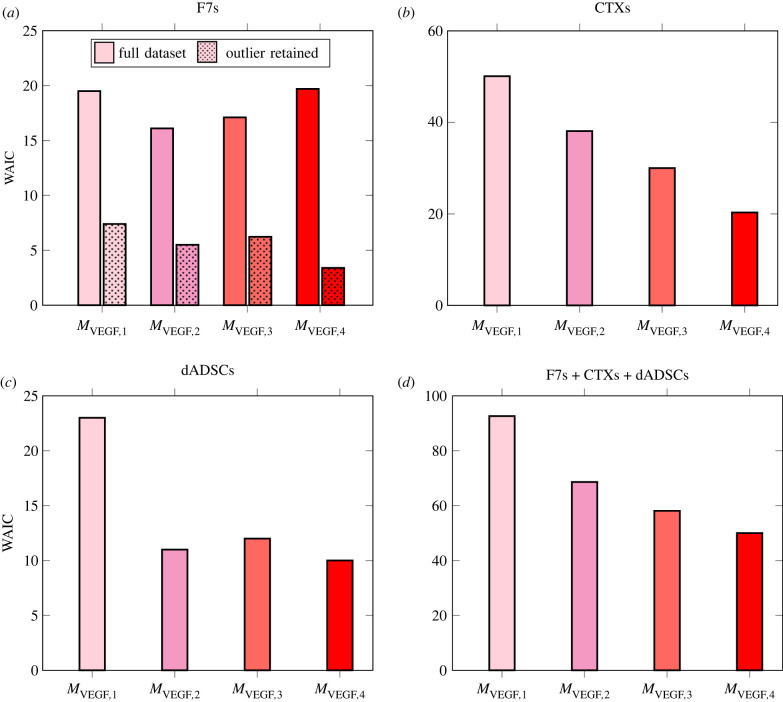
WAIC associated with each VEGF secretion model (*M*_VEGF,1_, *M*_VEGF,2_, *M*_VEGF,3_, *M*_VEGF,4_) for (*a*) F7 (*b*) CTX, (*c*) dADSC cells and (*d*) all three cell types together. (*a*) includes the WAIC associated with each VEGF secretion model for the case where a single outlier point ($C_{0,\mathrm{cell}}=60\times 10^{12}\;\mathrm{cell}\;m^{-3},C_{a/m}=3\%O_{2}$, see figure 7*l*) was excluded from calculation.

We use the posterior samples to compute the WAIC (equation (2.27)) associated with each VEGF secretion model, i.e. their ability to represent experimental data. Figure 5 shows the results for F7, CTX and dADSC. We see that for F7, all four models behave similarly, with *M*_VEGF,2_ performing the best. However, we see that when the influence of an outlier point not described by any model (*C*_0,cell_ = 60 × 10^12^ cell m^−3^, $C_{a/m}=3\%O_{2}$, see figure 7*l*) was excluded using an exaggerated uncertainty (10 times the value reported in table 10), the WAIC associated with each model decreases significantly, showing that the models are able to represent the remaining data, with *M*_VEGF,4_ performing better.

For CTX, we see that *M*_VEGF,4_ is associated with significantly lower WAIC and therefore is able to represent dataset. Finally, for dADSC we see that model *M*_VEGF,2_ behaves more poorly and *M*_VEGF,4_ performs better. Computing the sum of WAIC over all three cell types for the case of the complete dataset (figure 5*d*), we conclude *M*_VEGF,4_ is globally performing better. That is because *M*_VEGF,4_ is the only model to include nonlinear effects induced by local cell density through a crowding threshold (*C*_crowd_) that hinders cell secretion for larger cell densities. It also expresses VEGF secretion as a linear function of oxygen for large oxygen concentration ($\alpha ({\bar{C}}_{\mathrm{ox}}/C_{\mathrm{hypo}})$), while other models have a constant secretion rate at high oxygen concentration, or for the case of *M*_VEGF,1_, no secretion at all. Consequently, the latter fails to capture any data point lying above the upregulation threshold (*C*_hypo_), hence consistently ranking last.

#### 3.2.2. Experiment utility

Next we use the posterior samples to evaluate the Kullback–Leiber divergence (*D*_KL_, equation (2.31)). Doing so can be seen as the counterpart of computing the WAIC, as the latter evaluates the ability of a model to represent the data while the former evaluates the ability of an experimental design/dataset to inform a given model.

**Figure 6.**
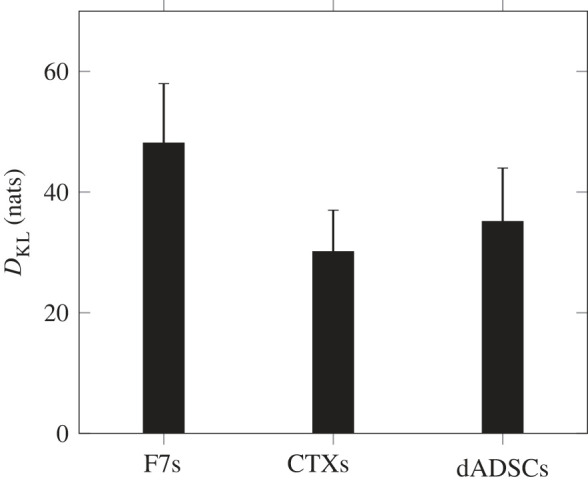
Kullback–Leiber divergence associated with each experimental dataset, i.e. each cell type (F7s, CTXs and dADSCs). Error bars correspond to the standard deviation associated with the VEGF secretion model (table 3).

Figure 6 shows the Kullback–Leiber divergence for each cell type. The values reported correspond to the average over the different VEGF secretion models and the error bars correspond to the associated standard deviations. We see that the information gain associated with F7 is larger than the one associated with CTXs, despite their underlying experimental designs being identical (table 2). This difference stems mostly from the experimental uncertainties being higher in the case of CTXs (table 10). Similarly, we see that the information gain associated with dADSCs is comparable to that associated with CTXs, despite its experimental design being larger (i.e. including more design points; cf. table 2). We attribute this to the much higher experimental uncertainties (appendix D) and the lack of glucose concentration measurement, which hinder the potential information gains.

#### 3.2.3. Pseudo-Bayesian model averaging

*Cell–solute interaction parameter*. Next, we use the WAIC estimated for each VEGF secretion model (figure 5) to derive the average posterior distribution (equation (2.28)) for each cell type, and deduce averaged posterior estimates for parameters shared across VEGF models. Table 5 shows $\bar{\mu_{\theta }}$ the average expected value, $\bar{\sigma_{\theta }}$ the average marginal standard deviation and $\bar{\left[\theta \right]}$ the average credible interval, for cell–solute interaction parameters. We can see that F7, CTX and dADSC cells exhibit different behaviours. In particular, we see that the oxygen maximum consumption rate (*M*_ox,max_) is the largest for F7 cells, which consume 2.5 times more oxygen than CTX cells and almost 12.5 times more than dADSCs.

**Table 5.** Posterior estimates for cell–solute interaction parameters. $\bar{\mu_{\theta }}$ corresponds to the expected value, $\bar{\sigma_{\theta }}$, to the standard deviation and $\bar{\left[\theta \right]}$ to the credible interval obtained using the averaged posterior distribution (equation (2.28)) while μ_*θ*_, *σ*_*θ*_ and [*θ*] correspond to the expected values, marginal standard deviation and credible intervals defined in equations (2.22), (2.23) and (2.24). Italic numbers correspond to the expected value estimates. Table 8 recapitulates the parameter descriptions.

parameter	units	F7s	CTXs	dADSCs
*M*_ox_ ($\bar{\mu_{\theta }}$, $\bar{\sigma_{\theta }}$, $\bar{\left[\theta \right]}$)				
*M*_ox,max_	10^−20^ kg s^−1^ cell^−1^	*11.89* 1.18 [10.42, 13.45]	*4.95* 0.73 [4.07, 5.90]	*0.95* 0.21 [0.70, 1.23]
*C*_ox,1/2_	4.21 × 10^−4^ kg m^−3^	*0.95* 0.23 [0.67, 1.23]	*0.87* 0.43 [0.33, 1.42]	*0.68* 0.32 [0.26, 1.11]
*M*_glu_ ($\bar{\mu_{\theta }}$, $\bar{\sigma_{\theta }}$, $\bar{\left[\theta \right]}$)				
*M*_glu,max_	10^−18^ kg s^−1^ cell^−1^	*7.82* 1.63 [5.72, 10.03]	*2.22* 0.89 [1.11, 3.43]	*5.31* 2.67 [1.80, 8.92]
*C*_glu,1/2_	kg m^3^	*0.55* 0.31 [0.15, 0.97]	*1.63* 0.78 [0.63, 2.65]	*0.87* 0.56 [0.17, 1.67]
*A*	n.a.	*0.88* 0.47 [0.35, 1.53]	*4.61* 2.41 [1.60, 7.96]	*3.94* 2.03 [1.24, 6.62]
*M*_cell_ ($\bar{\mu_{\theta }}$, $\bar{\sigma_{\theta }}$, $\bar{\left[\theta \right]}$)				
*γ*	10^−6^ s^−1^	*0.78* 0.52 [0.14, 1.51]	n.a.	*28.22* 8.10 [17.19, 38.34]
*C*_cell,max_	10^12^ cell m^−3^	*51.87* 16.42 [31.03, 72.82]	n.a.	144 94.32 [67.16, 229.32]
*δ*_0_	10^−6^ s^−1^	*0.35* 0.30 [0.05, 0.78]	*8.78* 1.13 [7.21, 10.33]	*7.90* 3.68 [3.16, 12.81]
*δ*_ox_	10^−6^ s^−1^	*0.72* 0.50 [0.15, 1.41]	*2.08* 1.10 [6.84, 5.78]	*4.48* 2.08 [1.72, 3.83]
*δ*_glu_	10^−6^ s^−1^	*1.71* 1.14 [0.36, 3.39]	*0.72* 0.32 [0.22, 1.23]	*1.78* 0.88 [0.62, 3.00]
*M*_VEGF,1_ (*μ*_*θ*_, *σ*_*θ*_, [*θ*])				
*β*	10^−22^ kg s^−1^ cell^−1^	*0.05* 0.02 [0.02, 0.07]	*0.57* 0.27 [0.25, 0.94]	*1.17* 0.53 [0.49, 1.89]
*C*_ox,hypo_	4.21 × 10^−4^ kg m^−^^3^	*1.33* 0.49 [0.75, 2.00]	*1.38* 0.47 [0.85, 2.05]	*7.5* 2.95 [4.05, 11.50]
*M*_VEGF,2_ (*μ*_*θ*_, *σ*_*θ*_, [*θ*])				
*α*	10^−24^ kg s^−1^ cell^−1^	*2.53* 1.19 [1.03, 4.16]	*10.61* 4.22 [4.34, 16.41]	*62.11* 27.01 [29.1, 97.9]
*V*	n.a.	*0.63* 0.30 [0.20, 1.00]	*2.43* 0.95 [1.28, 3.68]	*1.50* 1.03 [0.33, 2.97]
*ν*	n.a.	*3.88* 1.83 [1.41, 6.24]	*3.67* 1.81 [1.33, 6.10]	*3.28* 1.83 [0.88, 5.82]
*C*_ox,hypo_	4.21 × 10^−4^ kg m^−3^	*0.99* 0.55 [0.33, 1.73]	*1.01* 0.64 [0.32, 1.97]	*5.44* 2.83 [1.89, 9.25]
*C*_ox,hyper_	4.21 × 10^−4^ kg m^−3^	*4.77* 2.79 [1.20, 8.61]	*2.20* 2.22 [0.55, 5.51]	*11.90* 6.81 [3.58, 21.37]
*M*_VEGF,3_ (*μ*_*θ*_, *σ*_*θ*_, [*θ*])				
*α*_0_	10^−24^ kg s^−1^ cell^−1^	*2.61* 1.10 [1.01, 4.33]	*15.9* 6.04 [8.71, 24.01]	*57.03* 0.88 [21.33, 92.11]
*α*_1_	10^−38^ kg m^3^ s^−1^ cell^−2^	0.04 1.02 [− 1.28, 1.36]	*−0.24* 6.93 [− 9.14, 8.39]	*2.66* 2.05 [− 1.01, 5.39]
*α*_2_	10^−51^ kg m^6^ s^−1^ cell^−3^	*−0.01* 0.22 [− 0.28, 0.29]	*−0.70* 0.88 [− 1.82, 0.37]	*0.12* 0.10 [− 0.10, 0.30]
*V*_0_	n.a.	*0.55* 0.31 [0.16, 0.99]	*3.24* 1.24 [1.65, 4.88]	*1.92* 1.83 [0.24, 4.66]
*V*_1_	10^−14^ cell^−1^ m^3^	*0.83* 1.96 [− 1.64, 3.38]	*−0.35* 2.57 [− 3.47, 3.03]	*0.1* 0.1 [0.01 0.20]
*ν*	n.a.	*4.05* 1.84 [1.70, 6.57]	*3.86* 1.90 [1.51, 6.50]	*2.78* 1.83 [0.50, 5.20]
*C*_ox,hypo_	4.21 × 10^−4^ kg m^−3^	*1.08* 0.61 [0.35, 1.80]	*0.83* 0.35 [0.50, 1.23]	*5.88* 3.22 [1.32, 10.02]
*M*_VEGF,4_ (*μ*_*θ*_, *σ*_*θ*_, [*θ*])				
*α*	10^−24^ kg s^−1^ cell^−1^	*0.19* 0.11 [0.05, 0.35]	*1.12* 0.51 [0.44, 1.89]	*25.11* 10.70 [11.70, 39.40]
*β*	10^−22^ kg s^−1^ cell^−1^	*0.07* 0.03 [0.03, 0.10]	*2.54* 1.23 [1.12, 4.19]	*3.32* 1.92 [1.09, 5.90]
*C*_ox,hypo_	4.21 × 10^−4^ kg m^−3^	*1.08* 0.51 [0.80, 2.12]	*1.12* 0.44 [0.62, 1.73]	*5.94* 2.83 [2.54, 9.63]
*C*_cell,crowd_	10^12^cell m^−3^	*51.44* 22.04 [24.57, 80.36]	*26.67* 7.54 [18.53, 37.06]	*184.14* 102.11 [58.01, 325.11]

For glucose, maximum consumption rate (i.e. *M*_glu,max_(1 + *A*)), F7s and CTXs are comparable, but we note that F7 cells have a higher baseline glucose consumption rate. On the other hand, we see that dADSCs have a glucose maximum consumption rate approximately two times larger than F7s and CTXs, although we recall that no glucose measurement were included in the dADSC dataset, so this estimate is probably imprecise.

Regarding cell dynamics, we can see that CTX and dADSC cells have similar maximum death rates *Q*_max_ = *δ*_0_ + *δ*_ox_ + *δ*_glu_, i.e. the death rate associated with the asymptotic regime where oxygen and glucose are depleted. F7s, on the other hand, have a maximal death rate approximately five times smaller and therefore will deplete slower. What is more, F7s and dADSCs proliferate, whereas CTX cells are immortalized so do not. Looking at the asymptotic regime where oxygen and glucose are in excess, we can estimate the maximum proliferation rate as *P*_max_ = (1/2)(*γ* − *δ*_0_). We see that such a proliferation rate is 50 times larger for dADSCs than for F7s. Additionally, we note that in this regime we have, for both F7s and dADSCs, *δ*_0_ < *γ* so that there is a stable, steady-state cell density towards which the model will converge, i.e. ${\bar{C}}_{\mathrm{cell},\mathrm{stat}}=C_{\mathrm{cell},\max}(1-(\delta_{0}/\gamma )$) which is estimated at ${\bar{C}}_{\mathrm{cell},\mathrm{stat}}\approx 25\times 10^{12}\;\mathrm{cell}\;\;m^{-3}$ for F7s and ${\bar{C}}_{\mathrm{cell},\mathrm{stat}}\approx 104\times 10^{12}\;\mathrm{cell}\;\;m^{-3}$ for dADSC. We point out, however, that such a state only exists in asymptotic regimes and is degraded for lower oxygen and glucose concentration in the gel.

Finally, we see that all three cell types have widely different VEGF secretion rates, whether associated with baseline (*α*) or upregulated (*β* or *α*(1 + *V*)) states. In particular, we see that dADSC cells have a baseline VEGF secretion rate between 4 and 20 times larger than CTX cells and between 20 and 100 times larger than F7 cells, depending on the model considered. Similarly for the upregulated state, dADSCs secrete between 1.5 and 5 times more than CTXs and between 20 and 50 times more than F7s depending on the model considered.

*Transport parameters*. Next, similar to equation (2.28), which uses the WAIC as a proxy to create an averaged posterior distribution over the VEGF secretion models, we use the Kullback–Leiber divergence as a proxy to create an averaged distribution over the three cell types (replacing the minimum WAIC in equation (2.30) with the maximum *D*_KL_). We then apply this new averaging operator to the transport parameters that are shared across cell types, that were already averaged over the VEGF secretion models to obtain parameter estimates representative of the entire problem. The results are reported in table 6, which shows $\bar{\bar{\mu_{\theta }}}$ the expected value, $\bar{\bar{\sigma_{\theta }}}$ the marginal standard deviation and $\bar{\bar{\left[\theta \right]}}$ the credible interval averaged over both VEGF models and cell types.

**Table 6.** Posterior estimates for transport parameters. $\bar{\bar{\mu_{\theta }}}$ corresponds to the expected value, $\bar{\bar{\sigma_{\theta }}}$ the marginal standard deviation and $\bar{\bar{\left[\theta \right]}}$ the credible interval obtained by averaging across cell types and VEGF secretion models. Italic numbers correspond to the expected value estimates. Table 7 recapitulates the parameter descriptions.

parameter	units	$\bar{\bar{\mu_{\theta }}}$	$\bar{\bar{\sigma_{\theta }}}$	$\bar{\bar{\left[\theta \right]}}$
*D*_*m*,ox_	10^−9^ m^2^ s^−1^	*1.96*	0.06	[1.81,2.06]
*D*_*g*,ox_	10^−10^ m^2^ s^−1^	*4.51*	0.66	[3.64,5.36]
*η*_*g*/*m*,ox_	n.a.	*0.98*	0.02	[0.95,1.00]
*D*_*m*,glu_	10^−10^ m^2^ s^−1^	*9.56*	0.70	[8.64,10.49]
*D*_*g*,glu_	10^−10^ m^2^ s^−1^	*2.70*	0.32	[2.27,3.11]
*η*_*g*/*m*,glu_	n.a.	*1.40*	0.17	[1.13,1.61]
*D*_*m*,VEGF_	10^−10^ m^2^ s^−1^	*1.49*	0.10	[1.37,1.63]
*D*_*g*,VEGF_	10^−11^ m^2^ s^−1^	*4.96*	0.61	[4.15,5.74]
*η*_*g*/*m*,VEGF_	n.a.	*1.07*	0.12	[0.91,1.20]
*λ*_VEGF_	10^−5^ s^−1^	*7.05*	0.22	[3.84,10.46]

*Model predictions*. Next, we combine the values reported in tables 5 and 6 for cell–solute interactions and transport parameters, to the WAIC-average (i.e. average over the VEGF secretion model) of the initial and boundary conditions reported in electronic supplementary material (tables S1–S12) to simulate the different cell types in the well during 24 h.

**Figure 7.**
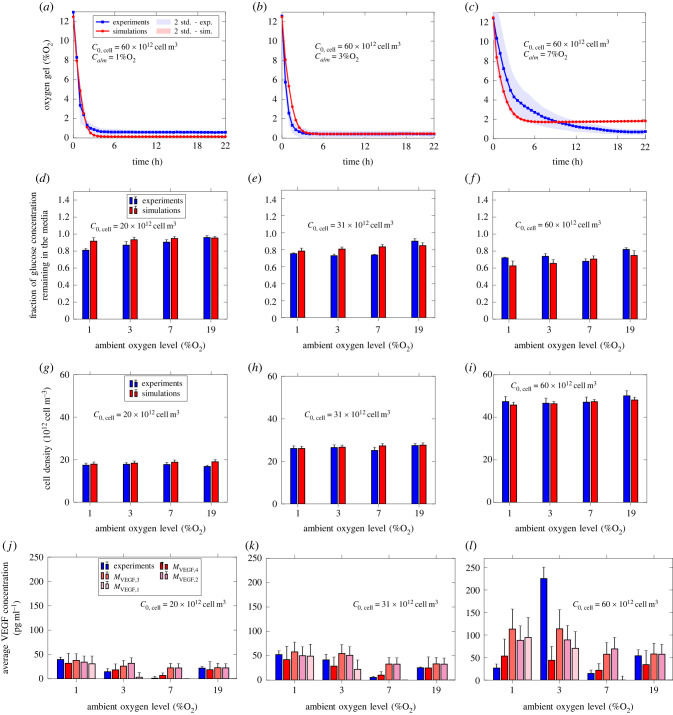
Comparison between experiment (blue) and simulation (red) for F7 cells. (*a*)–(*c*) Oxygen concentration in the gel. (*d*)–(*f*) Fraction of glucose concentration remaining in the media after 24 h. (*g*)–(*i*) Cell density in the gel after 24 h. (*j*)–(*l*) Mean VEGF concentration in the media after 24 h. Number of repeats for oxygen measurements *n* = 3. Number of repeats for glucose, cell and VEGF measurements *n* = 4. The red area (*a*–*c*) and error bars (*d*–*l*) correspond to the standard deviation associated with the model predictions based on the averaged posterior sample distribution. The blue area (*a*–*c*) and error bars (*d*–*l*) correspond to the standard deviation associated with the experiments.

**Figure 8.**
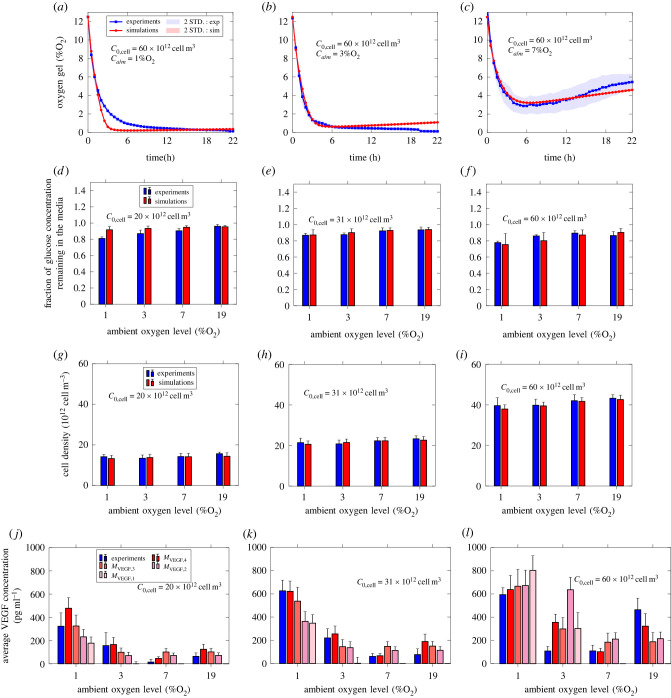
Comparison between experiment (blue) and simulation (red) for CTX cells. (*a*)–(*c*) Oxygen concentration in the gel. (*d*)–(*f*) Fraction of glucose concentration remaining in the media after 24 h. (*g*)–(*i*) Cell density in the gel after 24 h. (*j*)–(*l*) Mean VEGF concentration in the media after 24 h. Number of repeats for oxygen measurements *n* = 3. Number of repeats for glucose, cell and VEGF measurements *n* = 4. The red area (*a*–*c*) and error bars (*d*–*l*) correspond to the standard deviation associated with the model predictions based on the averaged posterior sample distribution. The blue area (*a*–*c*) and error bars (*d*–*l*) correspond to the standard deviation associated with the experiments.

**Figure 9.**
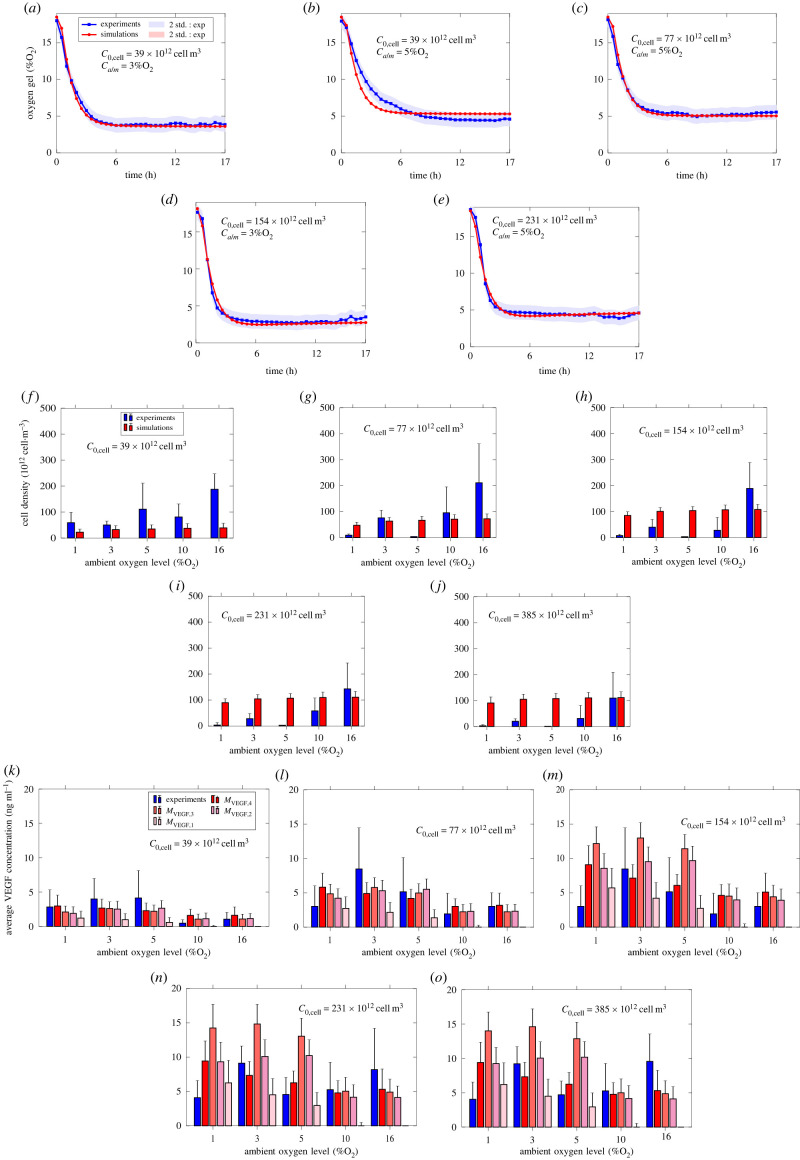
Comparison between experiment (blue) and simulation (red) for dADSC cells. (*a*)–(*e*) Oxygen concentration in the gel. (*f*)–(*j*) Cell density in the gel after 24 h. (*k*)–(*o*) Mean VEGF concentration in the media after 24 h. Number of repeats for oxygen measurements n.a. Number of repeats for cells and VEGF *n* ∈ [3 − 6]. The red area (*a*–*e*) and error bars (*f*–*o*) correspond to the standard deviation associated with the model predictions based on the averaged posterior sample distribution. The blue area (*a*–*e*) and error bars (*f*–*o*) correspond to the standard deviation associated with the experiments.

Figures 7, 8 and 9 compare the simulations (red) with the experiments (blue) for F7, CTX and dADSC cells, respectively. Figures 7, 8 and 9 all include the measured and simulated values for oxygen concentration in the gel, cell density in the gel and VEGF concentration in the media, while only figures 7 and 8 include the fraction of glucose concentration remaining in the media. Further, all three figures include the values produced for each VEGF model. Error bars for the experiments represent the standard deviations over the repeats. Error bars for the simulations correspond to the standard deviation associated with the model predictions based on the combined averaged posterior distributions.

Starting with F7 cells (figure 7), we see a good agreement for oxygen concentration, glucose concentration and cell density. For VEGF, we see that one point is poorly described, regardless of the model chosen. Given the moderate experimental uncertainties associated, this could indicate a gap in the model formulation. The general rationale underlying VEGF secretion models introduced in table 3 is that VEGF secretion is upregulated by low oxygen concentration, and hindered by increasing local cell density in the case of *M*_VEGF,4_. Such models are therefore inherently unable to capture such an isolated spike. One idea to overcome this could be to locally refine the experimental design (e.g. adding more initial cell densities) around the spike to potentially identify an undescribed metabolic process. Alternatively, surrogate model approaches such as Gaussian mixture model formulations [41] could be considered, with the trade-off of separating model formulation and metabolic processes description.

Similarly, figure 8 shows that simulations compare well with experiments in the case of CTX cells for oxygen, glucose, cell density and VEGF secretion.

For dADSC cells (figure 9), while we still see good agreement for oxygen, we note significant discrepancies for cell density. We link this to potential gaps in the model formulation, although some data points could also present some inconsistencies. For instance, figure 9*g*–*j* shows that there are cells surviving for $C_{a/m}=3\%O_{2}$ but not for $C_{a/m}=5\%O_{2}$, while figure 9*a*–*e* shows that the oxygen concentration in the gel remains consistent for such conditions regardless of the initial cell density. Additionally, cell density data are associated with high level of uncertainties compared with F7s and CTXs (table 10). Finally, we see that *M*_VEGF,4_ predictions are in good agreement with VEGF data, which is in line with its lower WAIC (figure 5), although here too we note that the data are associated with a larger standard deviation (table 10), which makes it difficult to highlight potential gaps in the models.

#### 3.2.4. Effect of information gain on parameters

**Figure 10.**
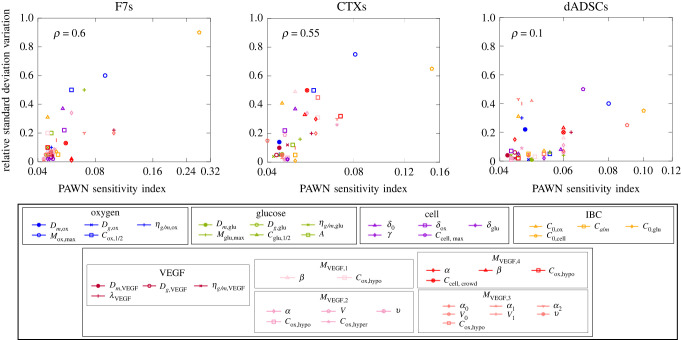
Marginal standard deviation relative variation ($\Delta \sigma_{\theta }=(\sigma_{\theta }^{\mathrm{prior}}-\sigma_{\theta }^{\mathrm{posterior}})/\sigma_{\theta }^{\mathrm{prior}}$) as a function of the PAWN sensitivity index, for oxygen (blue), glucose (green), cell (purple), initial and boundary conditions (orange) and VEGF (shades of red) related parameters, for F7, CTX and dADSC cells.

Figure 6 shows how informative the experimental datasets were for the models. An alternative way to look at the effect of information gain on individual parameters is to evaluate the relative variation of marginal standard deviation for each parameter, as a narrower distribution can be an indicator of information gain. We define such a variation as $\Delta \sigma_{\theta }=(\sigma_{\theta }^{{\;}_{\mathrm{prior}}}-\sigma_{\theta }^{{\;}_{\mathrm{posterior}}})/\sigma_{\theta }^{{\;}_{\mathrm{prior}}}$, where $\sigma_{\theta }^{{\;}_{\mathrm{prior}}}$ corresponds to the prior marginal standard deviation (appendix C.4) and $\sigma_{\theta }^{{\;}_{\mathrm{posterior}}}$ its posterior counterpart (tables 5 and 6). Figure 10 shows, for each parameter, Δ*σ*_*θ*_ as a function of their influence in the model output. We evaluate the global influence of a parameter using the median PAWN sensitivity indices (see appendix F), the larger the index, the greater the influence.

We can see that influential parameters are generally associated with larger marginal standard deviation variations, as illustrated by the correlation coefficient (*ρ*). This indicates that globally we tend to inform influential parameters preferentially. We also see that dADSCs are associated with a weaker correlation and a smaller mean variation in marginal standard deviation (11%) compared with F7 (18%) and CTX (20%) cells. This can be explained by the higher level of uncertainty associated with the experimental dataset (table 10). Additionally, glucose concentration was not measured for dADSCs, so glucose-related parameter marginal standard deviations (green symbols) are almost unaffected.

On the other hand, oxygen metabolism-related parameters, especially the maximum metabolic rate *M*_ox,max_ have their marginal standard deviation significantly reduced (60% for F7s, 75% for CTXs and 40% for dADSCs for *M*_ox,max_). Such parameters are influential as oxygen is directly involved in *M*_glu_, *M*_cell_ and *M*_VEGF_ , which may explain their variation in marginal standard deviation.

A similar conclusion can be drawn about baseline (*δ*_0_) and oxygen-related (*δ*_ox_) cell death rates for F7 and CTX cells, although we note that for dADSCs this effect is reduced due to the limited ability of the model to represent experimental data (figure 9*f*–*j*). Still we point out that the maximum cell density *C*_cell,max_ has its marginal standard deviation reduced by 50%. We attribute this to the large range of initial cell density (*C*_0,cell_) included in the experimental design which allows the triggering of crowding effects.

Finally, we see that the initial cell density (*C*_0,cell_) is the most influential parameter overall and has its marginal standard deviation significantly reduced across all three cell types (90% for F7s, 70% for CTXs and 40% for dADSCs). This is due to its high prior marginal standard deviation (appendix C) and role in controlling long-term cell density and, indirectly, cell metabolism. In comparison, the other initial and boundary conditions are generally less influential due to their smaller prior marginal standard deviation. Still, we note that the marginal standard deviation associated with the initial oxygen concentration *C*_0,ox_ is systematically reduced between 30 and 40% despite being marginally influential. That is because we have direct access to oxygen concentration measurements in the gel at early time points (cf. oxygen fields in figures 7–9).

Besides looking at the variations of marginal standard deviation for initial and boundary conditions, it is useful to look at how their posterior estimates compare with their prior counterparts. The latter, contrary to other parameters, are the result of experimental design, so that a strong variation might point toward potential experimental biases.

**Figure 11.**
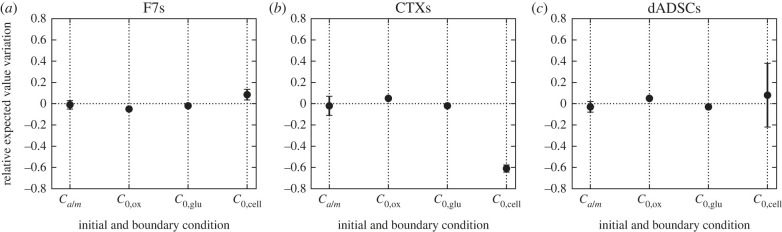
Expected value relative variation ($\Delta \mu_{\theta }=(\mu_{\theta }^{\mathrm{prior}}-\mu_{\theta }^{\mathrm{posterior}})/\mu_{\theta }^{\mathrm{prior}}$) for the oxygen concentration at the interface between air and media *C*_*a*/*m*_, the initial oxygen concentration *C*_0,ox_, the initial concentration in glucose *C*_0,glu_ and the initial cell density *C*_0,cell_. Error bars represents the standard deviation across the experimental plane (table 9).

In that sense, figure 11 shows the relative changes in the expected value for the initial and boundary conditions (i.e. oxygen concentration at the air/media interface *C*_*a*/*m*_, the initial oxygen concentration *C*_0,ox_, the initial glucose concentration *C*_0,glu_ and initial cell density *C*_0,cell_). Similar to the variation in marginal standard deviation, we define such a relative variation as $\Delta \mu_{\theta }=(\mu_{\theta }^{\mathrm{prior}}-\mu_{\theta }^{\mathrm{posterior}})/\mu_{\theta }^{\mathrm{prior}}$, where $\mu_{\theta }^{\mathrm{prior}}$ corresponds to the prior values reported in table 9 and $\mu_{\theta }^{\mathrm{posterior}}$ the estimates *a posteriori* obtained from electronic supplementary material (tables S1–S12). Similar to figure 10, the values were first averaged over the different VEGF secretion models. We also recall that each value of *C*_*a*/*m*_ and *C*_0,cell_ reported in table 9 is considered an independent parameter. In this context, the values displayed in figure 11 for these parameters are obtained by averaging Δμ_*θ*_ over all the values taken by such parameters. The error bars represent the associated standard deviation.

We see that *C*_*a*/*m*_, *C*_0,ox_ and *C*_0,glu_ remain generally close to their reported values (less than 10% change) regardless of the type of cell considered. This is a direct consequence of the small prior standard deviation, reflective of our higher confidence associated with such parameters. For *C*_*a*/*m*_ this result is reinforced by the asymptotic behaviour observed for dADSCs (figure 9*a*–*e*).

On the other hand, we see that the initial cell density (*C*_0,cell_) behaves differently. First, we see that for F7 cells the posterior estimates remain close to their prior estimate, with little spread. Combining this result with the 90% reduction in marginal standard deviation reported in figure 10 (yellow pentagons) suggests a correct initial estimation of the cell density.

For CTX cells, while we still observe little spread, we note that the posterior estimates are consistently larger (60%) than their prior reported values. Given that figure 10 also indicates a strong (70%) reduction in marginal standard deviation for this parameter, this could potentially point towards a prior underestimation of the initial cell density.

Finally, we see that for dADSCs the initial cell density remained unchanged on average but that the different estimates are significantly more spread than for F7s and CTXs, which is linked to the combined effect of the large prior marginal standard deviation and the large level of uncertainties in the dataset, especially for cell density (figure 9).

Figures 10 and 11 focus on the effect the information gain has on individual parameter, ignoring potential couplings emerging *a posteriori*. To address this, we compute the Pearson correlation coefficient matrix associated with the posterior parameter distribution

**Figure 12.**
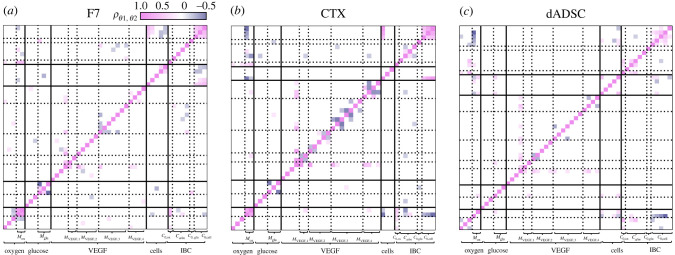
Pearson correlation coefficient $\rho_{\theta_{1},\theta_{2}}$ associated with the posterior distribution of transport, cell–solute and initial and boundary condition parameters for (*a*) F7, (*b*) CTX and (*c*) dADSC cells. Plain lines delimit the species considered (e.g. oxygen, glucose) and dotted lines represent the separation between transport and cell–solute interaction-related parameters, or between the different initial and boundary conditions. For clarity, correlation coefficients with $|\rho_{\theta_{1},\theta_{2}}|<0.2$ were rendered in white.

3.1 $$\rho_{\theta_{1},\theta_{2}}=\frac{1}{N_{s}}\frac{\sum_{i}((\theta_{i,1}-\mu_{\theta_{1}})(\theta_{i,2}-\mu_{\theta_{2}}))}{\sigma_{\theta_{1}}\sigma_{\theta_{2}}},$$

where *θ*_1_ and *θ*_2_ are any two parameters being inferred (tables 7–9). Figure 12 shows the Pearson correlation coefficient matrix for each cell type and each VEGF model. We see that for most parameters $|\rho_{\theta_{1},\theta_{2}}|<0.2$. We interpret this as no correlation, which we consider as evidence for the underlying hypothesis that all parameters in the problem are independent. The rest of the parameters were only weakly correlated, with no parameters having an absolute cross-correlation coefficient superior to 0.5, which can be considered as the threshold between weak and moderate correlation.

Figure 12 further shows that most non-zero correlation coefficients are the result of the coupling between cell–solute interaction parameters, i.e. parameters that underpin *M*_ox_, *M*_glu_, *M*_VEGF_ and *M*_cell_. This is not surprising, as cell–solute interaction terms can include explicit relationships between different species (e.g. *M*_cell_ (equation (2.16)) depends on oxygen concentration and glucose concentration) or even include parameters that are directly involved in other cell–solute interaction terms (e.g. *M*_cell_ (equation (2.16)) depends on *C*_ox,1/2_, which is also involved in *M*_ox_ (equation (2.14)) and *M*_glu_ (equation (2.15)) that favour correlation *a posteriori*. In addition, cell–solute interaction terms are composed of several parameters, each describing only a fraction of the underlying metabolic processes and can therefore be influenced by other parameters involved in describing the remainder of the processes.

On the other hand, transport parameters such as diffusion coefficients are seldom correlated with other parameters as they correspond to specific, independent processes.

Finally, figure 12 shows that initial and boundary condition parameters (*C*_0,ox_, *C*_*a/m*_, *C*_0,glu_, *C*_0,cell_) are also associated with non-zero Pearson correlation coefficients. First, we see that initial cell densities (*C*_0,cell_) are correlated between each other, regardless of cell type. This opens the possibility of modelling initial cell densities as coming from the same distribution, potentially decreasing the global parameter space. Then, initial and boundary condition parameters are correlated with different cell–solute interaction terms. Such couplings are mainly the effect of the nonlinear formulation of the cell–solute interaction terms (*M*_ox_, *M*_glu_, *M*_VEGF_ , *M*_cell_). This further motivates creating experimental design capable of taking advantage of the relationship between initial and boundary conditions and cell–solute interaction parameters.

#### 3.2.5. Experimental design

Figures 6, 10, 11 and 12 show the impact each experimental dataset has on the parameter posterior distributions. Such datasets originated from different experimental designs (table 2). These designs varied the oxygen concentration at the air/media interface (*C*_*a*/*m*_) and the initial cell density (*C*_0,cell_). In practice, other initial or boundary conditions could have been changed, i.e. initial oxygen concentration (*C*_0,ox_) and initial glucose concentration (*C*_0,glu_).

When devising a new experimental design to inform a model it can be difficult to evaluate which operating conditions should be prioritized. This is especially difficult for species such as VEGF, which are only indirectly controlled by the experimental conditions. We propose to use the Kullback–Leiber divergence (equation (2.31)) to determine which operating conditions should be varied in order to better inform model development.

To do so, we propose four experimental designs (table 12). Each design is centred around varying one initial or boundary condition, i.e. *C*_*a*/*m*_, *C*_0,ox_, *C*_0,glu_ and *C*_0,cell_. For each design, we calculate the associated Kullback–Leiber divergence (appendix G). We repeat the process for each VEGF secretion model reported in table 3.

**Figure 13.**
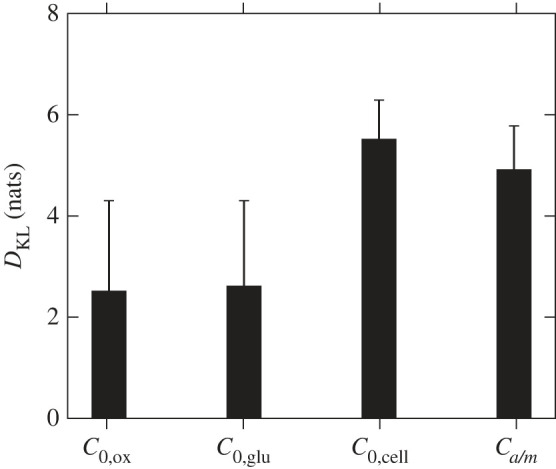
Kullback–Leiber divergence associated with each design of experiment presented in table 12. *x*-labels highlight the initial or boundary condition around which each experimental design is based on, i.e. initial oxygen concentration (*C*_0,ox_), initial glucose concentration (*C*_0,glu_), initial cell density (*C*_0,cell_) and oxygen concentration at the air/media interface (*C*_*a*/*m*_). High *D*_KL_ values infer a more informative design. Error bars represent the standard deviation over the four VEGF models reported in table 3.

Figure 13 shows the results obtained, with each point, similar to figure 6, corresponding to the average over the VEGF secretion models and error bars corresponding to the associated standard deviations for a given experimental design. We can see that designs centred around initial oxygen or glucose concentrations are less informative. For oxygen, this can be explained as the oxygen concentration profiles displayed in figures 7–9 show that the influence of initial oxygen concentration has relaxed after a few hours. As for initial glucose concentration, equations (2.9)–(2.18) and table 3 show that glucose concentration has only an indirect coupling with VEGF secretion, contrary to oxygen and cell density, and figure 10 shows that overall the influence of initial glucose concentration on the model output remains small.

On the other hand, we see that designs centred around initial cell density or air/media oxygen concentration are more informative. For the latter case, figures 7–9 show that *C*_*a*/*m*_ controls in part the long-term concentration of oxygen in the gel, which is a key component of each VEGF secretion model (table 3). Varying such a parameter then allows the models to exhibit both baseline and upregulated states, therefore yielding more information. As for initial cell density, VEGF secretion depends at least linearly on the cell density in the gel, which is still influenced by its initial value after 24 h as displayed by figures 7–9. What is more, *M*_VEGF,3_ and *M*_VEGF,4_ exhibit additional nonlinearities depending on the cell density. In this context, varying the initial cell density allows such behaviour to be highlighted and gain information.

Overall figure 13 points towards prioritizing initial cell density and air/media oxygen concentrations, with a slight preference for initial cell density when designing an *in vitro* experiment.

## 4. Discussion

Cellular engineered tissues have a high potential to improve nerve repair strategies. Optimizing the design of such tissues, however, represents a considerable challenge, given the large number of parameters available (therapeutic cell type, cell density, material density, arrangement, etc.), so that testing every combination possible using experiment in isolation is costly and time-consuming.

In this context, integrating a mathematical model to simulate nerve repair scenarios *in silico* can accelerate the development of such tissue (figure 1).

Being able to describe therapeutic cell behaviour in the engineered tissue is pivotal to deriving a model that is predictive of experimental scenarios. This process also offers the opportunity to investigate mechanisms underlying cell behaviour. This requires dedicated *in vitro* experiments in a controlled setting for a given cell type, which are seldom available. Performing systematic, extensive experiment campaigns can be practically infeasible due to time and cost constraints (e.g. new therapeutic cell types are constantly being considered [42,43]).

This results in experimental designs with limited resolution (e.g. small number of initial cell density considered) that themselves generate datasets associated with generally low spatial and temporal resolution (e.g. one measure of cell density the entire gel layer after 24 h). This is the source of experimental uncertainty. On the other hand, mathematical models rely on functional forms and parameters that need to be informed for the model to yield predictive power. The challenge for the model is then to maintain a subtle balance between the size and quality of the resulting experimental datasets and the number of parameters it uses, to avoid issues such as overfitting.

This work proposed to address this challenge by (i) building a pipeline to derive an experimentally informed mechanistic cell–solute model for therapeutic cell behaviour, accounting for experimental uncertainties (figure 2) and (ii) applying it to three therapeutic cell types (F7s, CTXs, dADSCs), for which dedicated *in vitro* experiments were performed (figure 3) so as to obtain a ready-to-use cell–solute model which allowed us to quantitatively compare different therapeutic cell types. Such a model could then be included in larger frameworks to explore nerve repair scenarios *in silico*, for instance by simulating cell–solute interactions in nerve conduits. This would offer the opportunity to compare the benefit of different conduit designs, in order to select the best performing one, e.g. the one associated with largest VEGF gradient or cell survival, to test *in vivo*.

Our approach relied on Bayesian inferences to parametrize the cell–solute model by combining prior knowledge of the parameters and likelihood to represent the new data and obtain posterior knowledge about them. We note that this approach is conditioned by the structure of the prior and likelihood distributions, as those reflect our knowledge of the problem. In this work, we used normal distributions as they are simple to interpret, rely only on a small number of parameters and are often used in other Bayesian frameworks [15,16], but we note that more complex distributions could have been used. We also point out that the prior estimates we used were based on previous estimates taken from the literature and asymptotic analysis (appendix C) so as to ensure being in a credible order of magnitude in the first place. Similarly, for the likelihood distribution we used the upper bound of the standard deviation associated with experimental repeats in order to mitigate overconfidence (appendix D).

Besides parameter estimation, we showed that the posterior distributions obtained could be used to discriminate between different functional forms using WAIC (figure 5), taking into account both quality of fit and number of parameters. We compared four VEGF secretion models (table 3) and concluded that *M*_VEGF,4_ was performing better overall, primarily due to its nonlinear account of local cell density through crowding effects. VEGF was selected in this work because it is central for the revascularization of the injury site and its secretion rate is generally not as understood as, for instance, oxygen. Still, we emphasize that our approach could be readily adapted to other cell–solute interactions (e.g. *M*_ox_, *M*_glu_ and *M*_cell_).

Mirroring model selection, the posterior distributions were also used to calculate the utility of each experimental dataset in informing the models, here defined as the Kullback–Leiber divergence (figure 6). It showed that the dataset associated with dADSCs was not the most informative, despite stemming from the largest experimental design (table 2), while the one associated with F7 cells was, primarily due to smaller uncertainties.

We then showed that WAIC and Kullback–Leiber divergence could be integrated into pseudo-Bayesian averaging to obtain summary parameter estimates representative of each model and each cell type.

Table 5 shows that the three cell types behave differently. Overall F7 cells consume oxygen at a higher rate so that they will probably create low-oxygen conditions sooner after the implantation of the repair construct and therefore potentially initiate regenerative angiogenesis sooner. What is more, they are associated with smaller death rates so that they would be able to sustain secreting VEGF over longer time scale. They are, however, associated with a much lower VEGF secretion rate than CTX and dADSC cells, so they might have a limited ability to build the strong gradient of VEGF necessary to trigger angiogenesis. On the other hand, dADSCs can sustain larger long-term cell density and secrete more VEGF, but have a much lower oxygen consumption rate, so will probably take more time to create the low-oxygen condition necessary to trigger the upregulation of VEGF secretion.

Parameter estimates in tables 5 and 6 could then be used as a basis for simulating cell–solute interactions in collagen gel. Figures 7–9 showed good agreement between experiments and simulations across all three species, which points toward capturing most of the mechanisms involved in cell behaviour at this scale. Still, there were some notable discrepancies, in particular for the description of dADSC density. Besides experimental uncertainties, such differences could stem from the limitations of logistic growth functional form we used to describe cell dynamics [44]. A following step could be to apply our approach to different cell dynamics relationships and select, using the WAIC, the most appropriate one to represent the data.

Beyond estimating representative parameter values, figures 10 and 11 highlighted the impact the information gain had on parameter distributions and showed that influential parameters tend to be informed more. In particular, figure 11 showed that the prior initial cell density estimates for CTX were potentially underestimated. Taken together, figures 10 and 11 show that combining relative variation in posterior marginal standard deviation ($\Delta \sigma_{\theta }$) and expected values ($\Delta \mu_{\theta }$) can be used to gain insight in the potential biases in the prior knowledge of the experimental conditions. Additionally, figure 12 showed that while most parameters remained independent of each other *a posteriori*, initial and boundary condition parameters were correlated with cell–solute interaction parameters, further highlighting the need for careful experimental design to inform the model.

Consistent with the strong influence of the initial cell density displayed in figures 10 and 13 showed that an experimental design including more initial cell density was more informative for the VEGF models and therefore should be prioritized in future experiments compared with experimental designs including more initial oxygen concentration, air/media oxygen concentration or initial glucose concentration. Still, we point out that the experimental designs tested were ranked according to how informative they were after 24 h. For longer periods, the oxygen concentration at the air/media interface will probably become more important as it is stationary, while the influence of initial conditions are likely to fade.

Additionally, the different experimental designs reported in table 12 were made simple on purpose in order to better highlight the impact of each initial and boundary conditions. In practice, however, a combination of them could be used. The next step would then be to extend this approach and to build a proper Bayesian experimental design [45], i.e. finding, for a fixed size, the combination of initial and boundary conditions that would be associated with the largest Kullback–Leiber divergence to optimize information gain for the model.

## 5. Conclusion

We built a pipeline capable of deriving experimentally informed cell–solute models to describe the behaviour of therapeutic cells used in nerve tissue engineering that involved balancing experimental uncertainties and model complexity. We applied it to three therapeutic cell types and deduced reference estimates which allowed us to quantitatively compare different candidate therapeutic cells. The model obtained could readily be used for *in silico* experiments of nerve repair scenarios, for instance to evaluate the benefit of a nerve conduit design.

Besides parameter estimations, we were able to interrogate models of VEGF secretion, and showed that both local cell density and local oxygen concentration should be considered, and subsequently selected the appropriate VEGF secretion model. We further explored the influence of experimental operating conditions on informing model parameters/behaviours and showed that initial cell density played a major role in informing the VEGF model secretion so that more initial cell densities should be included when designing future *in vitro* experiments.

Finally, this pipeline combines cell–solute modelling, Bayesian inferences and *in vitro* data for the first time in nerve tissue engineering, and is readily translatable to characterize the behaviour of other therapeutic cells in tissue engineering.

## Figures and Tables

**Figure 14.**
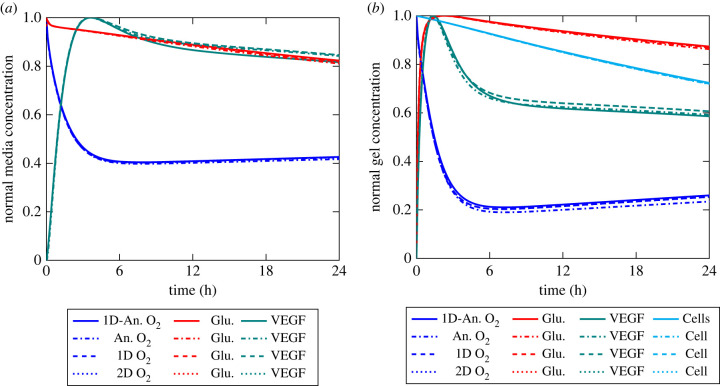
Comparison of normalized oxygen (blue), glucose (red), VEGF (green) concentration predictions and cell density (cyan) predictions between one-dimensional coarse mesh (plain), semi-analytical (dash-dotted), one-dimensional (dashed line) and two-dimensional (dotted) formulations in (*a*) the media and (*b*) the gel.

**Figure 15.**
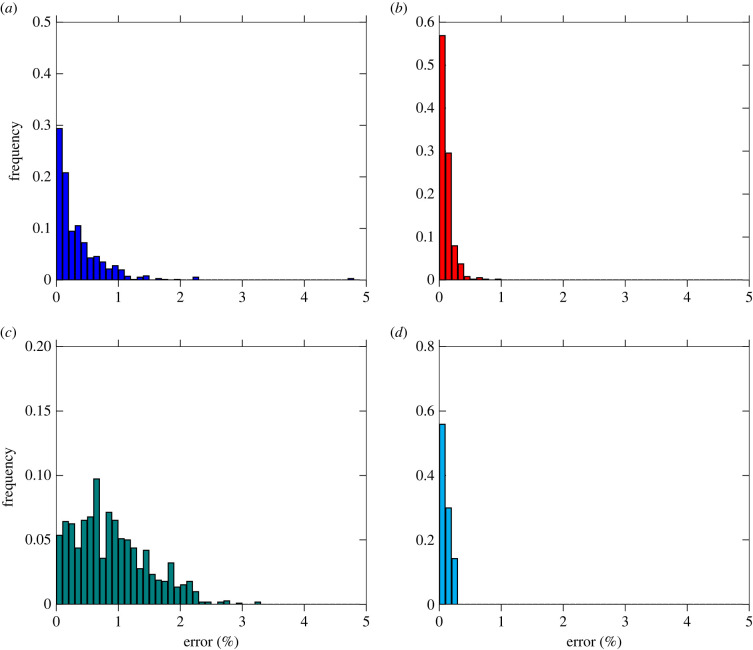
Distribution of relative differences between reference one-dimensional formulation and one-dimensional coarse mesh formulation for (*a*) oxygen concentration in the gel, (*b*) glucose concentration in the media, (*c*) VEGF concentration in the media and (*d*) cell density in the gel.

## Data Availability

All data are included in the manuscript and associated appendices/electronic supplementary material. Scripts and files can be access from the GitHub repository: https://github.com/MaximeTGB/TherapeuticCellsBehaviourInference. The data are provided in electronic supplementary material [46].

## Appendix A. Effective transport equations

**A.1. Culture media**

Exploiting the rotational symmetry highlighted in figure 3*a,b* in equation (2.1) yields

A 1 $$\partial_{t}C_{m}=D_{m}\left(\frac{1}{r}\partial_{r}(r\partial_{r}C_{m})+\partial_{z}^{2}C_{m}\right)-\lambda C_{m},$$

with *r* and *z* denoting the radial and axial coordinates, respectively. We non-dimensionalize equation (A 1) with

$$r=(R_{a/m}-R_{g/m})\rho ,\;z=L_{m}\xi ,\;t=\frac{L_{m}^{2}}{D_{m}}\tau ,$$

where *L*_*m*_ is the thickness of the media layer and *R*_*a*/*m*_ and *R*_*g*/*m*_ are the radii of the well at the air/media and gel/media interfaces, respectively. This yields

A 2 $$\partial_{\tau }C_{m}=R^{\prime -2}\frac{1}{\rho }\partial_{\rho }(\rho \partial_{\rho }C_{m})+\partial_{\xi }^{2}C_{m}-DaC_{m},$$

where *R*′ = −(*R*_*a*/*m*_ − *R*_*g*/*m*_)/*L*_*m*_ corresponds to the slope of the well and $Da=\lambda L_{m}^{2}/D_{m}$ to the Damköhler number associated with degradation. Assuming *R*′ ≪ 1, we expand the concentration field in powers of the small well slope parameter through

A 3 $$C_{m}=C_{m,0}+R^{\prime }C_{m,1}+R^{\prime 2}C_{m,2}\cdots$$

Additionally, we will assume that *Da* = *O*(*R*′^−1^), i.e. that the reaction is much stronger than the diffusion. We do so since the resulting effective one-dimensional equation has the same form as for *Da* = *O*(1) but is less straightforward to obtain. Finally, we assume that $\partial_{\tau }\cdot =\partial_{T}\cdot +Da\partial_{ℵ}\cdot$ to be able to capture the variations associated with diffusion as well as degradation. Doing so allows us to write the following sequence of equations:

A 4 $$O(R^{\prime -2})\;0=\frac{1}{\rho }\partial_{\rho }(\rho \partial_{\rho }C_{m,0}),$$

A 5 $$O(R^{\prime -1})\;R^{\prime }Da\partial_{ℵ}C_{m,0}=\frac{1}{\rho }\partial_{\rho }(\rho \partial_{\rho }C_{m,1})-R^{\prime }DaC_{m,0}$$

A 6 $$\begin{array}{r} \mathrm{and}\;O(1)\;\partial_{T}C_{m,0}+R^{\prime }Da\partial_{ℵ}C_{m,1}=\frac{1}{\rho }\partial_{\rho }(\rho \partial_{\rho }C_{m,2})+\partial_{\xi }^{2}C_{m,0}\\ -R^{\prime }DaC_{m,1}. \end{array}$$

A similar expansion can be applied to the boundary condition at the well wall noting

A 7 $$n\cdot \nabla C_{m}=0\;⟺\;-R^{\prime -2}\partial_{\rho }C_{m}=\partial_{\xi }C_{m},$$

which leads to

A 8 $$O(R^{\prime -2})\;\partial_{\rho }C_{m,0}=0,$$

A 9 $$O(R^{\prime -1})\;\partial_{\rho }C_{m,1}=0$$

and

A 10 $$O(1)\;\partial_{\rho }C_{m,2}=-\partial_{\xi }C_{m,0}.$$

We then combine equations (A 4)–(A 10) to obtain an effective one-dimensional effective equation. To do so, we define the following averaging operator:

A 11 $$\langle \cdot \rangle =\frac{1}{\pi R^{2}}\int_{0}^{2\pi }\int_{0}^{R}\cdot rdrd\theta =2{\left(\frac{R_{a/m}-R_{g/m}}{R}\right)}^{2}\int_{0}^{R/(R_{a/m}-R_{g/m})}\cdot \rho \;d\rho ,$$

where *θ* corresponds to the rotation angle and *R*(*z*) = *R*′ *z* + *R*_*a*/*m*_ to the local radius of the well. Applying the averaging operator to equation (A 4) while taking into boundary condition (A 8) yields

A 12 $$C_{m,0}(\rho ,\xi ,\tau )=C_{m,0}(\xi ,\tau ),$$

hence we have 〈*C*_*m*,0_〉 = *C*_*m*,0_(*ξ*, *τ*). Doing the same with equation (A 5) while taking into account boundary condition (A 9), leads to

A 13 $$R^{\prime }Da\langle \partial_{ℵ}C_{m,0}\rangle =-R^{\prime }Da\langle C_{m,0}\rangle .$$

The above equation can be simplified since $\langle \partial_{ℵ}C_{m,0}\rangle =\partial_{ℵ}\langle C_{m,0}\rangle =\partial_{ℵ}C_{m,0}$. Equation (A 5) now reads

A 14 $$\;0=\frac{1}{\rho }\partial_{\rho }(\rho \partial_{\rho }C_{m,1}).$$

Applying the averaging operator on the above equation taking into account boundary conditions (A 9) again leads to

A 15 $$C_{m,1}(\rho ,\xi ,\tau )=C_{m,1}(\xi ,\tau ),$$

so that 〈*C*_*m*,1_〉 = *C*_*m*,1_(*ξ*, *τ*). Applying the averaging operator to equation (A 6), taking into account boundary condition (A 10), noticing that $\left\langle \partial_{\xi }^{2}C_{m,i}\rangle =\partial_{\xi }^{2}\langle C_{m,i}\right\rangle$ for *i* = 0, 1 because we are in a conic geometry, and 〈∂_*T*_ *C*_*m*,0_〉 = ∂_*T*_〈*C*_*m*,0_〉 and $\left\langle \partial_{ℵ}C_{m,1}\rangle =\partial_{ℵ}\langle C_{m,1}\right\rangle$, this leads to

A 16 $$\partial_{T}\langle C_{m,0}\rangle +R^{\prime }Da\partial_{ℵ}\langle C_{m,1}\rangle =-2\frac{R_{a/m}-R_{g/m}}{R}\partial_{\xi }\langle C_{m,0}\rangle +\partial_{\xi }^{2}\langle C_{m,0}\rangle -R^{\prime }Da\langle C_{m,1}\rangle .$$

To close the problem, we assume that

A 17 $$\langle C_{m}\rangle =\langle C_{m,0}\rangle ,$$

so that 〈*C*_*m*,*i*_〉 = 0, *i* > 0. Consequently, equation (A 16) now reads

A 18 $$\partial_{T}\langle C_{m,0}\rangle =-2\frac{R_{a/m}-R_{g/m}}{R}\partial_{\xi }\langle C_{m,0}\rangle +\partial_{\xi }^{2}\langle C_{m,0}\rangle .$$

Finally, recalling that $\partial_{T}\langle C_{m,0}\rangle =\partial_{\tau }\langle C_{m,0}\rangle -Da\langle \partial_{ℵ}C_{m,0}\rangle$, and that we have from equation (A 13) $Da\langle \partial_{ℵ}C_{m,0}\rangle =-Da\langle C_{m,0}\rangle$, equation (A 18) becomes

A 19 $$\partial_{\tau }\langle C_{m}\rangle =-2\frac{R_{a/m}-R_{g/m}}{R}\partial_{\xi }\langle C_{m}\rangle +\partial_{\xi }^{2}\langle C_{m}\rangle -Da\langle C_{m}\rangle ,$$

which corresponds to the target effective transport equation. We recall that the derivation is done for the case *Da* = *O*(*R*′^−1^) but the same form is still valid for *Da* = *O*(1). For smaller Damköhler scalings (e.g. *O*(*R*′)), the only change is the simplification of the last term in equation (A 19). To avoid loss of generality, we are going to keep equation (A 19) in the next sections, which has the following expression once dimensionalized:

A 20 $$\partial_{t}\langle C_{m}\rangle =2D_{m}\frac{R^{\prime }}{R}\partial_{z}\langle C_{m}\rangle +D_{m}\partial_{z}^{2}\langle C_{m}\rangle -\lambda \langle C_{m}\rangle .$$

**A.2. Interface air/media**

At the air/media interface, the boundary condition is either a Dirichlet or a Neumann condition depending on the species considered. For a Dirichlet boundary condition, the transformation is straightforward,

A 21 $$\langle C_{m}\rangle =C_{a/m}.$$

For a Neumann boundary condition, this yields

A 22 $$-D_{m}\partial_{z}\langle C_{m}\rangle =0,$$

neglecting *O*(*R*′^2^) terms.

**A.3. Interface gel/media**

At the gel/media interface continuity of mass flux is imposed (equation (2.7)) so that

A 23 $$-D_{m}\partial_{z}\langle C_{m}\rangle =\frac{1}{S_{g/m}}\iint_{S_{g/m}}-D_{g}\partial_{z}C_{g}dS_{g/m},$$

and the thermodynamic equilibrium

A 24 $$\lim_{t\to \infty }\langle C_{m}\rangle =\frac{\eta_{g/m}}{S_{g/m}}\iint_{S_{g/m}}C_{g}dS_{g/m}.$$

**A.4. Collagen gel**

We integrate equation (2.2) over the gel domain so that

A 25 $${∭}_{\Omega_{g}}\partial_{t}C_{g}d\Omega =\iint_{\partial \Omega_{g}}n\cdot D_{g}\nabla C_{g}d\partial \Omega +\iint_{\Omega_{g}}M(C_{g})d\Omega .$$

Taking into account the Neumann boundary conditions at the well walls and bottom, the above equation becomes

A 26 $${∭}_{\Omega_{g}}\partial_{t}C_{g}\;d\Omega =\iint_{S_{g/m}}-D_{g}\partial_{z}C_{g}\;dS_{g/m}+\iint_{\Omega_{g}}M(C_{g})\;d\Omega .$$

Since we consider the gel layer to be thin we are going to assume that the volume-averaged concentration is representative of the concentration within the gel so that the above equation reads

A 27 $$d_{t}{\bar{C}}_{g}=\frac{1}{\Omega_{g}}\iint_{S_{g/m}}-D_{g}\partial_{z}C_{g}dS_{g/m}+M({\bar{C}}_{g}),$$

where ${\bar{C}}_{g}=(1/\Omega_{g}){∭}_{\Omega_{g}}C_{g}d\Omega$ is the average concentration in the gel. Further applying the conservation of mass (equation (A 23)) this yields

A 28 $$d_{t}{\bar{C}}_{g}=-\frac{S_{g/m}}{\Omega_{g}}D_{m}\partial_{z}\langle C_{m}\rangle +M({\bar{C}}_{g}).$$

To close the problem, we then to use the following membrane boundary condition that satisfies equation (A 24):

A 29 $$-D_{m}\partial_{z}\langle C_{m}\rangle =K(\langle C_{m}\rangle -\eta_{g/m}{\bar{C}}_{g}),$$

where *K* represents an effective permeability *K* = *D*_*g*_/*L*_*g*_. Equation (A 28) then becomes

A 30 $$d_{t}{\bar{C}}_{g}=\frac{S_{g/m}K}{\Omega_{g}}(\langle C_{m}\rangle -\eta_{g/m}{\bar{C}}_{g})+M({\bar{C}}_{g}).$$

## Appendix B. Numerical methods

Equations (2.9)–(2.13) form a nonlinear system that we solve iteratively using finite volume methods. Since the resolution of such a problem is combined to Monte Carlo Markov chains, a number of simulations are going to be performed. Therefore, we use a coarse mesh and a large time step to reduce computational costs. We describe the gel with a unique mesh cell and the media with a few other so that the total number of mesh cells typically lies below 10. To mitigate the effect of having a coarser mesh, we based the estimation of the differential operators at the interface between mesh cells using analytical solutions, instead of the usual approximations, assuming that the concentration profile in between two mesh cell centres in the media reached stationary state during one time step so that we can write

B 1 $$2D_{m}\frac{R^{\prime }}{R_{i}+R^{\prime }\zeta }\partial_{z}C_{\left[i,i+1\right]}+D_{m}\partial_{z}^{2}C_{\left[i,i+1\right]}-\lambda C_{\left[i,i+1\right]}=0,$$

B 2 $$C_{\left[i,i+1\right]}=C_{i}^{k+1},\;\zeta =0$$

and

B 3 $$C_{\left[i,i+1\right]}=C_{i+1}^{k+1},\;\zeta =\Delta z,$$

where *C*_[*i*,*i*+1]_(*ζ*) represents the concentration profile in between mesh cells *i* and *i* + 1, *ζ* the local axial position, and $C_{i}^{k+1}$ and $C_{i+1}^{k+1}$ the concentration at the centre of mesh cells at time step *k* + 1. While equation (B 1) holds in each mesh cell located in the media, the local boundary conditions (equations (B 2) and (B 3)) can be changed to accommodate for the global boundary conditions (equations (2.10)–(2.12)) where necessary. Equation (B 1) has the following homogeneous solutions:

B 4 $$C_{\left[i,i+1\right]}(\zeta )=A\frac{e^{-\sqrt{\lambda /D_{m}}\zeta }}{R_{i}+R^{\prime }\zeta }+B\frac{e^{\sqrt{\lambda /D_{m}}\zeta }}{2\sqrt{\lambda /D_{m}}(R_{i}+R^{\prime }\zeta )},\;\lambda >0$$

and

B 5 $$C_{\left[i,i+1\right]}=A+B\frac{1}{R_{i}+R^{\prime }\zeta },\;\lambda =0,$$

where *A* and *B* are constants that are computed using the local boundary conditions (equations (B 2) and (B 3) and their variants). In general, *C*_[*i*,*i*+1]_ can be written under the form

B 6 $$C_{\left[i,i+1\right]}(\zeta )=f_{i}(\zeta )C_{i}^{k+1}+f_{i+1}(\zeta )C_{i+1}^{k+1},$$

where *f*_*i*_ and *f*_*i*+1_ are functions deduced from *A* and *B*. This solution can then be used in the evaluation of the differential operator at the interface between the two mesh cells.

We note that for the asymptotic case of pure diffusion in a cylinder, i.e. *λ* = 0, *R*′ → 0, equation (B 6) writes *C*_[*i*,*i*+1]_ = (1 − (*ζ*/Δ*z*))*C*_*i*_ + (*ζ*/Δ*z*)*C*_*i*+1_ and has the following gradient ∂_*ζ*_ *C*_[*i*,*i*+1]_ = (*C*_*i*_ − *C*_*i*+1_)/Δ*z* which corresponds to the order 1 approximation used in most classical numerical methods when solving diffusion.

Implementation was done in Python using standard packages (Numpy 1.18.1 and Scipy 1.4.1). Simulations were run on a desktop computer with intel Xeon W 18-core 2.3 GHz and 128 GB Ram 2666 MHz DDR4. Time resolution of the cell–solute problem for one specific set of values for initial and boundary condition was 0.01 s.

To verify this coarse-mesh approximation, we compared it against a classic, refined, one-dimensional finite volume resolution of equations (2.9)–(2.13). We also included a comparison with a two-dimensional axisymmetrical finite volume resolution of the initial set of equations (equations (2.1)–(2.8)) and a comparison with a semi-analytical solution of equations (2.9)–(2.13) we derived based on the superposition of Sturm–Liouville eigenvalue problems. While elegant, such a solution is quite sensitive to the number of eigenvalues chosen so that it quickly becomes computationally comparable to a refined one-dimensional approach and was not selected to be used for MCMC.

Figure 14 shows the normalized average concentration in the media (figure 14*a*) and gel (figure 14*b*) as a function of time for the four resolution methods aforementioned using parameter values listed in tables 7–9 and VEGF secretion model *M*_VEGF,4_ for the case of F7 cells. We see that refined one-dimensional and two-dimensional cases are superimposed, validating our derivation of equations (2.9)–(2.13). Then, we see that both the semi-analytical and coarse-mesh approximations generally agree well with the two reference solutions. Figure 14, compares the models only for one specific set of parameters. To have a better idea of the general behaviour of the coarse-mesh approximation, we sampled intervals derived from the prior distribution using Morris trajectories [47]. For each sample, we compared the one-dimensional reference with the one-dimensional coarse predictions, amounting to around 10 000 simulations in total, which figure 15 summarizes. In particular, we computed the relative difference in (i) the average concentration of oxygen in the gel (figure 15*a*), (ii) the average concentration of glucose in the media (figure 15*b*), (iii) the average concentration of VEGF in the media (figure 15*c*), and (iv) the average cell density in the gel (figure 15*d*) after 24 h. We can see that the coarse-mesh approximation lies within 2% of the one-dimensional reference solution in general, except for VEGF where it lies within 4%, which we consider acceptable for the purpose of inverse problem resolution.

**Table 7.** Description and prior estimation for transport parameters.

parameter	units	description	value
*D*_*m*,ox_	10^−9^ m^2^ s^−1^	diffusion coefficient of oxygen in the media	2.60
*D*_*g*,ox_	10^−10^ m^2^ s^−1^	diffusion coefficient of oxygen in the gel	7.00
*η*_*g*/*m*,ox_	n.a.	gel/media partition coefficient for oxygen	1.00
*D*_*m*,glu_	10^−10^ m^2^ s^−1^	diffusion coefficient of glucose in the media	9.60
*D*_*g*,glu_	10^−10^ m^2^ s^−1^	diffusion coefficient of glucose in the gel	2.70
*η*_*g*/*m*,glu_	n.a.	gel/media partition coefficient for glucose	1.50
*D*_*m*,VEGF_	10^−10^ m^2^ s^−1^	diffusion coefficient of VEGF in the media	1.50
*D*_*g*,VEGF_	10^−11^ m^2^ s^−1^	diffusion coefficient of VEGF in the gel	5.00
*η*_*g*/*m*,VEGF_	n.a.	gel/media partition coefficient for VEGF	1.10
*λ*_VEGF_	10^−5^ s^−1^	VEGF degradation	5.00

**Table 8.** Description and prior estimation for cell–solute interaction parameters.

parameter	units	description	F7s	CTXs	dADSCs
*M*_ox,max_	10^−20^ kg s^−1^ cell^−1^	maximum oxygen metabolic rate	5.00	5.00	0.60
*C*_ox,1/2_	4.21 × 10^−4^ kg m^−3^	concentration at half the maximum metabolic rate	0.80	1.50	0.50
*M*_glu,max_	10^−18^ kg s^−1^ cell^−1^	maximum glucose metabolic rate	6.20	1.80	4.00
*C*_glu,1/2_	kg m^3^	concentration at half the maximum metabolic rate	0.50	1.40	0.95
*A*	n.a.	anaerobic glucose consumption factor	1.80	4.60	3.20
*γ*	10^−6^ s^−1^	cell proliferation rate	0.90	n.a.	22.00
*C*_cell,max_	10^12^ cell m^−3^	maximum cell density sustaining cell proliferation	30.00	n.a.	400.00
*δ*_0_	10^−6^ s^−1^	baseline cell death rate	1.20	3.20	5.30
*δ*_ox_	10^−6^ s^−1^	oxygen-related cell death rate	3.90	2.50	3.20
*δ*_glu_	10^−6^ s^−1^	glucose-related cell death rate	2.50	0.60	1.50
c*M*_VEGF,1_					
*β*	10^−22^ kg s^−1^ cell^−1^	upregulated VEGF secretion rate	0.04	0.90	1.10
*C*_ox,hypo_	4.21 × 10^−4^ kg m^−3^	upregulation threshold	1.00	1.10	4.80
*M*_VEGF,2_					
*α*	10^−24^ kg s^−1^ cell^−1^	baseline VEGF secretion rate	2.90	12.00	50.00
*V*	n.a.	transition coefficient	1.50	2.00	2.00
*ν*	n.a.	transition coefficient	3.00	3.00	3.00
*C*_ox,hypo_	4.21 × 10^−4^ kg m^−3^	upregulation threshold	1.00	1.10	4.80
*C*_ox,hyper_	4.21 × 10^−4^ kg m^−3^	baseline threshold	5.00	5.00	10.00
*M*_VEGF,3_					
*α*_0_	10^−24^ kg s^−1^ cell^−1^	baseline VEGF secretion rate	2.90	12.00	50.00
*α*_1_	10^−38^ kg m^3^ s^−1^ cell^−2^	baseline secretion rate order 1 deviation coefficient	0.00	0.00	0.00
*α*_2_	10^−51^ kg m^6^ s^−1^ cell^−3^	baseline secretion rate order 2 deviation coefficient	0.00	0.00	0.00
*V*_0_	n.a.	baseline VEGF secretion rate	0.50	2.00	2.00
*V*_1_	10^−14^ cell^−1^ m^3^	upregulation order 1 deviation coefficient	0.00	0.00	0.00
*ν*	n.a.	transition coefficient	3.00	3.00	3.00
*C*_ox,hypo_	4.21 × 10^−4^ kg m^−3^	upregulation threshold	1.00	1.10	4.80
*M*_VEGF,4_					
*α*	10^−24^ kg s^−1^ cell^−1^	baseline VEGF secretion rate	0.29	1.20	22.00
*β*	10^−22^ kg s^−1^ cell^−1^	upregulated VEGF secretion rate	0.05	2.90	4.00
*C*_ox,hypo_	4.21 × 10^−4^ kg m^−3^	upregulation threshold	1.00	1.10	4.80
*C*_cell,crowd_	10^12^ cell m^−3^	crowding threshold	60.00	23.00	200.00

**Table 9.** Description and prior estimation for initial and boundary condition parameters.

parameter	units	description	F7s	CTXs	dADSCs
*C*_*a*/*m*_	4.21 × 10^−4^ kg m^−3^	oxygen concentration at the air/media interface	1/3/7/19	1/3/7/19	1/3/5/10/16
*C*_0,ox_	4.21 × 10^−4^ kg m^−3^	initial oxygen concentration	12.5	12.5	18.5
*C*_0,glu_	kg m^−3^	initial glucose concentration	4.5	4.5	4.5
*C*_0,cell_	10^12^ cell m^−3^	initial cell density	20/31/60	20/31/60	39/77/154/231/385

## Appendix C. Prior distribution parameters

**C.1. Transport parameters**

We consider the culture media to behave very similarly to water. In that respect, we use reference values for the diffusion coefficient in the media (*D*_*m*_) for oxygen [48], glucose [49,50] and VEGF [33] in water at 37°C, respectively.

The stabilized gel, on the other hand is composed of approximately 5–10% collagen [20], and between approximately 1.5% and 15% cells in volume (depending on the initial cell density) so that we expect the diffusion coefficient in the gel (*D*_*g*_) to be smaller than in the media. We use Stokes–Einstein Law to obtain a first approximation of the diffusion coefficient, which falls reasonably close to the range of values reported in the literature for oxygen [13,51], glucose [13,52] and VEGF [13,53,54].

Since the gel is primarily composed of water, we can expect the concentration jump resulting from the compounded effect of chemical affinity and porous structure represented by the partition coefficient (*η*_*g*/*m*_) to be small, so that continuity condition (i.e. *η*_*g*/*m*_ = 1) remains reasonable, in particular for small molecules such as oxygen. For glucose and VEGF, we use values evaluated in the layer of the skin (glucose) [55] and the extracellular matrix of muscle tissue (VEGF) [56]. Finally, we estimate the degradation rate for VEGF (*λ*_VEGF_) using values from the literature [8,13,54,57].

**C.2. Cell–solute interaction parameters**

This work is, to the best of our knowledge the first where F7 cells are being characterized in such a controlled environment. Consequently, there is little information available with regard to the value of cell–solute interaction parameters associated with these cells. To avoid using non-informative prior, we combine a simplified version of equations (2.9)–(2.18) and table 3 with the measures reported in figure 7*a*,*b* to obtain a crude approximation of the mean of the prior distribution of each parameter.

Starting with oxygen-related parameters, we consider the cases *C*_0,cell_ = 6 × 10^13^ cell m^−3^ and $C_{a/m}=1\%O_{2}$ and $C_{a/m}=3\%O_{2}$, for which figure 7 shows that oxygen levels measured in the gel are approximately constant after a few hours. Considering that this constant value is the result of the balance between the oxygen flux coming from the air/media interface and the consumption of oxygen in the gel yields

C 1 $$M_{\mathrm{ox},\max}\approx \frac{D_{m,\mathrm{ox}}D_{g,\mathrm{ox}}S_{g/m}(C_{a/m}-{\bar{C}}_{\mathrm{ox},S})({\bar{C}}_{\mathrm{ox},S}+C_{\mathrm{ox},1/2})}{{\bar{C}}_{\mathrm{cell},T}{\bar{C}}_{\mathrm{ox},S}\Omega_{g}(L_{g}D_{m,\mathrm{ox}}+L_{m}D_{g,\mathrm{ox}})},$$

where ${\bar{C}}_{\mathrm{cell},T}$ is the cell density after 24 h and ${\bar{C}}_{\mathrm{ox},S}$ is the asymptotic value of oxygen concentration in the gel. The above equation should yield for both $C_{a/m}=1\%O_{2}$ and $C_{a/m}=3\%O_{2}$ so that we can deduce

C 2 $$C_{\mathrm{ox},1/2}\approx \frac{\kappa_{1\%O_{2}}}{\kappa_{3\%O_{2}}-\kappa_{1\%O_{2}}}{\bar{C}}_{\mathrm{ox},S,1\%O_{2}}-\frac{\kappa_{3\%O_{2}}}{\kappa_{3\%O_{2}}-\kappa_{1\%O_{2}}}{\bar{C}}_{\mathrm{ox},S,3\%O_{2}},$$

where $\kappa =(C_{a/m}-{\bar{C}}_{\mathrm{ox},S})/{\bar{C}}_{\mathrm{cell},T}{\bar{C}}_{\mathrm{ox},S}$.

A comparable rationale can be applied to cell dynamics. We consider the same cases, i.e. *C*_0,cell_ = 6 × 10^13^ cell m^−3^ and $C_{a/m}=1\%O_{2}$ and $C_{a/m}=3\%O_{2}$ for which we already know that oxygen levels in the gel are low, and for which crowding effects are likely to be high. In this context, cell proliferation is hindered, hence *P* ≈ 0. Additionally, we see that the concentration of glucose in the media remains high after 24 h. Since the Damköhler number associated with glucose consumption in the gel $Da_{\mathrm{glu}}=L_{g}^{2}M_{\mathrm{glu},\max}C_{0,\mathrm{cell}}/D_{g,\mathrm{glu}}C_{0,\mathrm{glu}}$ is small, it suggests that the glucose concentration in the gel also remains high during the first 24 h. As consequence, we can assume that glucose-related cell deaths are only a fraction of baseline and oxygen-related cell deaths and can be ignored. This yields

C 3 $$\delta_{0}+\delta_{\mathrm{ox}}\frac{C_{\mathrm{ox},1/2}}{{\bar{C}}_{\mathrm{ox},S}+C_{\mathrm{ox},1/2}}\approx \frac{1}{T}\ln\left(\frac{C_{0,\mathrm{cell}}}{{\bar{C}}_{\mathrm{cell},T}}\right),$$

where *T* represents a day in seconds. Following previous estimation for CTXs, and to be consistent with our hypothesis, we further evaluate *δ*_glu_ ≈ 1/2(*δ*_0_ + *δ*_ox_). Next, we evaluate proliferation-related parameters using the case $C_{a/m}=19\%O_{2}$ and $C_{0,\mathrm{cell}}=2\times 10^{13}\;\mathrm{cell}\;m^{-3}$, i.e. high oxygen concentration and low cell density, so that oxygen is probably in excess and crowding effects are probably weak. This leads to $P\approx \gamma {\bar{C}}_{\mathrm{cell}}$ and $Q\approx \delta_{0}{\bar{C}}_{\mathrm{cell}}$. In this context, we deduce that

C 4 $$\gamma \approx \delta_{0}-\frac{1}{T}\ln\left(\frac{C_{0,\mathrm{cell}}}{{\bar{C}}_{\mathrm{cell},T}}\right).$$

Finally, it is possible to estimate *C*_cell,max_. To do so, we consider the case $C_{a/m}=19\%O_{2}$ and *C*_0,cell_ = 6 × 10^13^ cell m^−3^, i.e. high oxygen concentration and high initial cell density, so oxygen is probably in excess and cell crowding effects are probably maximal, hence we assume $P\approx \gamma {\bar{C}}_{\mathrm{cell}}(1-({\bar{C}}_{\mathrm{cell}}/C_{\mathrm{cell},\max}))$ and $Q\approx \delta_{0}{\bar{C}}_{\mathrm{cell}}$. In this context, we can write

C 5 $$C_{\mathrm{cell},\max}\approx C_{0,\mathrm{cell}}\frac{\gamma }{\left(\delta_{0}-\gamma \right)}\frac{\left(e^{(\delta_{0}-\gamma )T}-1\right)}{\left((C_{0,\mathrm{cell}}/{\bar{C}}_{\mathrm{cell},T})-e^{(\delta_{0}-\gamma )T}\right)}.$$

We then apply the same rationale to glucose. We consider the case $C_{a/m}=19\%O_{2}$ and *C*_0,cell_ = 2 × 10^13^ cell m^−3^, i.e. high oxygen concentration and low initial cell density, so that impact of anaerobic respiration is minimal. Additionally, we consider glucose to remain in excess in the gel due to the Damköhler number being small, hence $M_{\mathrm{glu}}\approx -M_{\mathrm{glu},\max}{\bar{C}}_{\mathrm{cell}}$. In this context, we deduce that

C 6 $$M_{\mathrm{glu},\max}\approx \frac{\Omega_{m}}{\Omega_{g}}\frac{\left(C_{0,\mathrm{glu}}-{\bar{\left\langle C\right\rangle }}_{\mathrm{glu},T}\right)(\gamma -\delta_{0})}{C_{0,\mathrm{cell}}(e^{(\gamma -\delta_{0})T}-1)},$$

where ${\bar{\left\langle C\right\rangle }}_{\mathrm{glu},T}$ corresponds the average concentration of glucose in the media after 24 h and $\Omega_{m}$ the volume of the media. Similarly, we consider the case $C_{a/m}=1\%O_{2}$ and $C_{0,\mathrm{cell}}=6\times 10^{13}\;\mathrm{cell}\;m^{-3}$, i.e. low oxygen concentration and high initial cell density, so that anaerobic consumption is promoted. In this context, we deduce that

C 7 $$A\approx \frac{\Omega_{m}}{\Omega_{g}}\frac{\left(C_{0,\mathrm{glu}}-{\bar{\left\langle C\right\rangle }}_{\mathrm{glu},T}\right)(\gamma -\delta_{0}-\delta_{\mathrm{ox}}(C_{\mathrm{ox},1/2}/({\bar{C}}_{\mathrm{ox},S}+C_{\mathrm{ox},1/2})))}{M_{\mathrm{glu},\max}C_{0,\mathrm{cell}}(e^{(\gamma -\delta_{0}-\delta_{\mathrm{ox}}(C_{\mathrm{ox},1/2}/({\bar{C}}_{\mathrm{ox},S}+C_{\mathrm{ox},1/2})))T}-1)}-1.$$

We consider the concentration in the media to be high, and since the Damköhler number associated with glucose metabolism is small, we also consider the concentration in the gel to be high, so that glucose is in excess and the associated Michaelis–Menten kinetics is saturated. This requires *C*_glu,1/2_ to be small compared with the concentration of glucose in the gel, hence we assume *C*_glu,1/2_ ≈ 0.1 *C*_0,glu_.

Finally, we apply the same reasoning to VEGF secretion. We consider the case $C_{a/m}=19\%O_{2}$, *C*_0,cell_ = 2 × 10^13^ cell m^−3^ so that oxygen is likely to be in excess, hence $M_{\mathrm{VEGF}}\approx \alpha {\bar{C}}_{\mathrm{cell}}$. In this context, we have

C 8 $$\alpha \approx \frac{\Omega_{m}{\bar{\left\langle C\right\rangle }}_{\mathrm{VEGF},T}(\gamma +\lambda -\delta_{0})}{\Omega_{g}C_{0,\mathrm{cell}}(e^{(\gamma -\delta_{0})T}-e^{-\lambda T})},$$

where ${\bar{\left\langle C\right\rangle }}_{\mathrm{VEGF},T}$ corresponds to the average VEGF concentration in the media after 24 h. Next, we consider the case $C_{a/m}=1\%O_{2}$, *C*_0,cell_ = 6 × 10^13^ cell m^−3^ so that VEGF secretion is upregulated by low-oxygen condition, hence $M_{\mathrm{VEGF}}\approx \beta {\bar{C}}_{\mathrm{cell}}$. In this context, we have

C 9 $$\beta \approx \frac{\Omega_{m}{\bar{\left\langle C\right\rangle }}_{\mathrm{VEGF},T}(\lambda -\delta_{0}-\delta_{\mathrm{ox}}(C_{\mathrm{ox},1/2}/({\bar{C}}_{\mathrm{ox},S}+C_{\mathrm{ox},1/2})))}{\Omega_{g}C_{0,\mathrm{cell}}(e^{(-\delta_{0}-\delta_{\mathrm{ox}}(C_{\mathrm{ox},1/2}/({\bar{C}}_{\mathrm{ox},S}+C_{\mathrm{ox},1/2})))T}-e^{-\lambda T})}.$$

We note that this procedure treats baseline and upregulated secretion rates as parameters of a generic secretion model; however, they can then be used as the basis to evaluate parameters specific to each VEGF secretion model reported in table 3.

Starting with *M*_VEGF,1_ we have $\alpha_{M_{\mathrm{VEGF}},1}\approx 0$ and $\beta_{M_{\mathrm{VEGF},1}}\approx \beta$. We then infer from the experimental values reported in figures 7–9, that $C_{\mathrm{ox},\mathrm{hypo},M_{\mathrm{VEGF}},1}\approx 1\%O_{2}$.

For *M*_VEGF,2_, we have $\alpha_{M_{\mathrm{VEGF}},2}\approx \alpha$ and *V* ≈ *β*/*α* − 1. Similarly, we have $C_{\mathrm{ox},\mathrm{hypo},M_{\mathrm{VEGF}},2}\approx 1\%O_{2}$ and $C_{\mathrm{ox},\mathrm{hyper},M_{\mathrm{VEGF}},2}\approx 5\%O_{2}$. Finally, we follow [33] and have $\nu_{M_{\mathrm{VEGF}},2}\approx 3$.

For *M*_VEGF,3_, we have *α*_0_ ≈ *α*. We treat *α*_1_ and *α*_2_ as higher-order deviations, so that *α*_1_ = *α*_2_ = 0. Similarly, we have *V*_0_ ≈ *β*/*α* − 1 and *V*_1_ = 0. We then have $C_{\mathrm{ox},\mathrm{hypo},M_{\mathrm{VEGF}},3}\approx 1\%O_{2}$ and similar to *M*_VEGF,2_ we assume $\nu_{M_{\mathrm{VEGF}},3}\approx 3$.

For *M*_VEGF,4_, finally, we have $\alpha_{M_{\mathrm{VEGF},4}}\approx \alpha$ and $\beta_{M_{\mathrm{VEGF},4}}\approx \beta$. Again we have $C_{\mathrm{ox},\mathrm{hypo},M_{\mathrm{VEGF}},4}\approx 1\%O_{2}$. As for the cell crowding effect, we have $C_{\mathrm{cell},\mathrm{crowd}}\approx 6\times 10^{13}\;\mathrm{cell}\;m^{-3}$, as figure 7 shows that VEGF secretion does not seem hindered by higher cell density.

We note that the above procedure can be also be applied to CTXs and dADSCs. When doing so, values fall reasonably close to the ones found in [8,13] for which dedicated experiments were performed or within range of values found in the literature for other types of cell (e.g. $M_{\mathrm{ox},\max}\in [10^{-20},5\times 10^{-17}]\;\mathrm{kg}\;s^{-1}\;{\mathrm{cell}}^{-1}$ [8,13,58,59], *M*_glu,max_ ∈ [10^−18^, 4 × 10^−17^] kg s^−1^ cell^−1^ [13,60,61]).

We point out, however, that only *M*_VEGF,3_ and *M*_VEGF,4_ were parametrized in [8] and [13] for CTXs and dADSCs, respectively. Therefore, all other VEGF secretion-related parameters are estimated using the above procedure. We finally note that [8] proposes to use $\nu_{M_{\mathrm{VEGF},3}}\approx 405$ for dADSCs, but since tanh(3)/tanh(405) ≈ 0.99 we consider it unnecessary to explore such high values and will consider $\nu_{M_{\mathrm{VEGF},3}}\approx 3$ for the case of dADSCs as well.

We also recall that no glucose measurements were available for dADSC so no glucose-related parameters were estimated in [8]. However, we note that values estimated for CTXs and F7s were comparable for glucose-related parameters. Since CTXs, F7s and dADSCs all mimic Schwann cell phenotypes, we assume dADSCs glucose-related parameters to be the average of the value estimated for CTXs and F7s.

**C.3. Initial and boundary condition parameters**

For all initial and boundary conditions, the mean value is taken as the reported experimentally controlled value, and the standard deviation is set based on our confidence in that value. Beginning with the ambient (*C*_*a*/*m*_) and initial (*C*_0,ox_) oxygen levels in the gel and initial glucose concentration (*C*_0,glu_), were straightforward to control so were assigned a relatively small standard deviation $\sigma_{\theta }^{\mathrm{prior}}=0.1\mu_{\theta }^{\mathrm{prior}}$ (i.e. 10% of the value reported experimentally). On the other hand, the initial cell density (*C*_0,cell_) is obtained after gel compression, a step with the potential to introduce additional variability, so a larger standard deviation ($\sigma_{\theta }^{\mathrm{prior}}=0.5\mu_{\theta }^{\mathrm{prior}}$) was imposed. Similarly, initial oxygen level in the media, which was not directly measured, is also associated with a larger marginal standard deviation ($\sigma_{\theta }^{\mathrm{prior}}=0.5\mu_{\theta }^{\mathrm{prior}}$).

**C.4. Marginal standard deviation**

We use the marginal standard deviation as a proxy for the confidence we have in the prior knowledge of a given parameter. To keep things tractable, we use $\sigma_{\theta }^{\mathrm{prior}}=0.5\mu_{\theta }^{\mathrm{prior}}$, $\sigma_{\theta }^{\mathrm{prior}}=0.1\mu_{\theta }^{\mathrm{prior}}$ or $\sigma_{\theta }^{\mathrm{prior}}=0.05\mu_{\theta }^{\mathrm{prior}}$ where *X*, for each cell type, represents any transport or cell solute interaction parameter or initial and boundary condition.

The case $\sigma_{\theta }^{\mathrm{prior}}=0.5\mu_{\theta }^{\mathrm{prior}}$ allows for the exploration within the same order of magnitude and is used for parameters that have been estimated using the procedure described in appendix C.2, that are scarcely represented in the literature or that cannot be controlled finely (cf. appendix C.3). This concerns all parameter reported in table 8 as well as the initial concentration of oxygen (*C*_0,ox_) and the initial cell density (*C*_0,cell_).

The case $\sigma_{\theta }^{\mathrm{prior}}=0.1\mu_{\theta }^{\mathrm{prior}}$, on the other hand, allows for the exploration of smaller deviations and is used for parameters better established in the literature or parameters that can be controlled finely (cf. appendix C.3). This includes the initial glucose concentration (*C*_0,glu_), the oxygen concentration at the interface between air and media (*C*_*a*/*m*_) and the diffusion coefficients in the gel (*D*_*g*,ox_, *D*_*g*,glu_, *D*_*g*,VEGF_ , *η*_*g*/*m*,ox_, *η*_*g*/*m*,glu_, *η*_*g*/*m*,VEGF_).

Finally, the case $\sigma_{\theta }^{\mathrm{prior}}=0.05\mu_{\theta }^{\mathrm{prior}}$ is used specifically for the diffusion coefficients in the media, as those are well established.

The only exceptions to these rules are *α*_1_, *α*_2_ and *V*_1_, which are treated as deviations of the baseline secretion rate in *M*_VEGF,3_. We set their standard deviation so that $\sigma_{\alpha_{1}}^{\mathrm{prior}}\approx 0.1\alpha_{0}/C_{0,\mathrm{cell}}$ and $\sigma_{\alpha_{2}}^{\mathrm{prior}}\approx 0.1\alpha_{0}/C_{0,\mathrm{cell}}^{2}$. Similarly, we have $\sigma_{V_{1}}^{\mathrm{prior}}\approx 0.1(V_{0}/C_{0,\mathrm{cell}})$. We use an initial cell density of 40 × 10^12^ cell m^−3^ for CTX and F7 cells and 200 × 10^12^ cell m^−3^, as those correspond to approximately the middle of the range of cell density explored in each case.

## Appendix D. Likelihood distribution parameters

We estimate the experimental data point value *Y*_*k*_ as the mean over the experimental repeats (blue curves and bar in figures 4 and 7 to 9) for each species and for each cell type.

We then estimate the marginal standard deviation associated with each data point *σ*_e,*k*_. Instead of using the pointwise standard deviation associated with experimental repeats (i.e. the error bars reported in figures 4–9 for each data point) we rather consider the upper bound associated with each dataset, i.e. $\sigma_{e,X}=\max_{1\leq k\leq N_{e}}\sigma_{e,X,k}$ with *X* ∈ {oxygen, glucose, VEGF, cell} in order to mitigate overconfidence. Table 10 recapitulates the values used for *σ*_e,*X*_ for each cell type.

**Table 10.** Standard devitation (*σ*_*e*_) associated with the noise used to describe the likelihood distribution associated with the different species.

specie	units	F7s	CTXs	dADSCs
oxygen concentration in the gel (${\bar{C}}_{\mathrm{ox}}$)	4.21 × 10^−4^ kg m^−3^	0.6	0.4	0.5
fraction of glucose remaining in the media (${\bar{\left\langle C\right\rangle }}_{\mathrm{glu}}/C_{0,\mathrm{glu}}$)	n.a.	0.03	0.05	n.a.
average concentration of VEGF in the media (${\bar{\left\langle C\right\rangle }}_{\mathrm{VEGF}}$)	10^−9^ kg m^3^	50	150	3000
living cell density in the gel ${\bar{C}}_{\mathrm{cell}}$	10^12^ cell m^−3^	3	5	100

When repeats were not available, e.g. for the measures of oxygen concentration in the gel in the acellular and dADSC cell cases, we assumed a standard deviation corresponding to the average of the ones used for F7 and CTX cells, again, to avoid overconfidence.

## Appendix E. Convergence of the Monte Carlo Markov chains and robustness of the predictions

We sample the posterior distribution using Monte Carlo Markov chains (MCMC) based on a Metropolis–Hastings Algorithm [62], for each VEGF model and each cell type approximately 10^6^ times, assuming normal state transition coefficients for each of the parameters reported in tables 7–9. Transition coefficients were chosen so that approximately 40% of the proposals were accepted to ensure reasonable mixing of the chain. They were generally of the order of 5% of prior marginal standard deviation. Precisely, we ran 10 MCMCs in parallel, each starting from a random point sampled from the prior distribution. The simulations were run on a desktop computer with intel Xeon W 18-core 2.3 GHz and 128 GB Ram 2666 MHz DDR4. Time resolution of the cell–solute problem for one specific set of values for initial and boundary condition was 0.01 s. Typical time resolution for one of the experiment designs introduced in table 2 was 0.1 s. Time resolution for a single MCMC chain sampling was 24 h. Implementation of the Metropolis–Hasting algorithm was done in Python using standard packages (Numpy 1.18.1 and Scipy 1.4.1).

In order to estimate the statistics defined in §2.3.4, MCMCs have to be converged. MCMCs are indeed stochastic by nature and exhibit a transient regime during which ergodicity is not respected. To overcome this, we discard the first 20% of the sample distribution that is called the burn-in. We then assess if the remaining distribution is converged by adapting the *Z*-value derived by Geweke [63], which we defined as

E 1 $$Z_{\theta }=\frac{\left|\mu_{\theta ,\alpha }-\mu_{\theta ,\beta }\right|}{\sqrt{\sigma_{\theta ,\alpha }^{2}+\sigma_{\theta ,\beta }^{2}}},$$

where *α* and *β* correspond to two intervals of length $N_{\alpha }$ and $N_{\beta }$. We used the first 10% and last 50% of the chain after removing the burn-in and checked if their difference in average lay within 2 standard deviations. Table 11 shows that it is the case for every parameters for F7s and VEGF model *M*_VEGF,4_.

**Table 11.** ${\mathrm{GRS}}_{\theta }$, $Z_{\theta }$ and $\phi_{\theta }$ for each parameter, for the case of F7s and VEGF model *M*_VEGF,4_. values reported for *C*_*a*/*m*_ and for *C*_0,cell_ are averaged values over the different conditions reported in table 2.

parameter	${\mathrm{GRS}}_{\theta }$	$Z_{\theta }$	$\phi_{\theta }$
transport			
*D*_*m*,ox_	1.01	0.05	0.07
*D*_*g*,ox_	1.03	0.03	0.01
*η*_*g*/*m*,ox_	1.05	0.10	0.10
*D*_*m*,glu_	1.01	0.02	0.05
*D*_*g*,glu_	1.04	0.04	0.15
*η*_*g*/*m*,glu_	1.05	0.18	0.03
*D*_*m*,VEGF_	1.03	0.06	0.10
*D*_*g*,VEGF_	1.13	0.03	0.05
*η*_*g*/*m*,VEGF_	1.07	0.05	0.15
*λ*_VEGF_	1.06	0.07	0.26
initial and boundary condition			
*C*_*a*/*m*_	1.10	0.10	0.11
*C*_0,ox_	1.11	0.09	0.53
*C*_0,glu_	1.01	0.01	0.05
*C*_0,cell_	1.08	0.08	0.47
cell–solute interaction			
*M*_ox,max_	1.03	0.04	0.05
*C*_ox,1/2_	1.15	0.01	0.10
*M*_glu,max_	1.06	0.15	0.30
*C*_glu,1/2_	1.03	0.09	0.23
*A*	1.02	0.14	0.15
*γ*	1.04	0.10	0.16
*C*_cell,max_	1.08	0.04	0.24
*δ*_0_	1.01	0.02	0.26
*δ*_ox_	1.05	0.03	0.51
*δ*_glu_	1.03	0.08	0.44
*α*	1.03	0.06	0.12
*β*	1.02	0.03	0.29
*C*_ox,hypo_	1.05	0.05	0.06
*C*_cell,crowd_	1.02	0.10	0.13

Besides assessing the convergence of each chain individually, we also assess the convergence in between the chains run in parallel. To do so we computed the Gelman–Rubin statistic (GRS) [64] defined the following way:

E 2 $${\mathrm{GRS}}_{\theta }=\sqrt{\frac{N_{s}-1}{N_{s}}+\frac{N_{\mathrm{chain}}}{N_{\mathrm{chain}}-1}\frac{\sum_{n}{\left(\mu_{\theta }^{\mathrm{chain},n}-{\bar{\mu }}_{\theta }^{\mathrm{chain}}\right)}^{2}}{\sum_{n}{\left(\sigma_{\theta }^{\mathrm{chain},n}\right)}^{2}}},$$

where *N*_chain_ denotes the number of Markov chains used, $\mu_{\theta }^{\mathrm{chain},n}$ and $\sigma_{\theta }^{\mathrm{chain},n}$ the mean and standard deviation of a parameter *θ* within chain *n* and where ${\bar{\mu }}_{\theta }^{\mathrm{chain}}$ represents the mean of a parameter *θ* across all chains. The idea behind this criterion is that, using chains with different starting points, they should converge to the same final distribution so that the GRS should converge to 1. Table 11 shows the results for 10 chains for the case of F7 cells and VEGF model *M*_VEGF,4_. As we can see in tables GRS < 1.2 regardless of the parameter, which we consider as evidence of converging behaviour.

Additionally, the solutions of the inverse problem presented in the Results section are based on all available data. To assess the robustness of the parameter estimation and model fitting, we adapt *k*-fold cross validation. Briefly, we separate the experiment dataset into *k* components, leave one of the components out and perform Bayesian inference using the remaining *k* − 1 components. We repeat the process once for each of the components. The measurements included in each component *k* are picked randomly, without repetition. The number of measurements varies between oxygen, VEGF, cell density and glucose, so each species was partitioned independently. In this work, we choose *k* = 5, i.e. 20% of the dataset is retained. We then compute the mean absolute difference between expected values obtained using partial and complete datasets and compare it with the posterior marginal standard deviation obtained using the complete dataset, i.e.

E 3 $$\phi_{\theta }=\frac{1}{N_{k}}\sum_{k=1}^{k=5}\frac{\left|\mu_{\theta }^{\mathrm{partial},k}-\mu_{\theta }^{\mathrm{complete}}\right|}{\sigma_{\theta }^{\mathrm{complete}}},$$

where *N*_*k*_ represents the number of partition, here 5. Table 11 presents the results for the case of F7s and VEGF secretion model *M*_VEGF,4_. We see that the expected values obtained using a partial dataset remain close to the ones obtained using the complete dataset and are in general within 50% of the posterior marginal standard deviation, which points towards a consistent estimation of the parameters. Finally, we compute the relative variation in WAIC (equation (2.27)). We find, on average less than 10% change between partial and complete dataset cases, indicating very similar ability to represent the data.

## Appendix F. PAWN sensitivity analysis

We quantify the influence a given parameter has on the model output using the PAWN sensitivity analysis [65]. This method is a global sensitivity analysis which produces summary statistics based on the model output cumulative density function. Briefly, the idea is to calculate a distance between the unconditional cumulative distribution associated with the model output and the conditional cumulative distribution for which one parameter is fixed. The larger the distance between the two distributions the more influential the parameter. This distance evaluation is repeated for different values of the parameter fixed (referred to as conditioning intervals) and a summary statistic is chosen to represent the distribution of distances obtained. Here we consider the median distance to mitigate effects of extreme values. This median is referred to as the median PAWN sensitivity index.

To evaluate such indices, parameters have to be sampled multiple times. To do so we create intervals based on prior distribution so that $I_{\theta ,j}=[\mu_{\theta ,j}^{\mathrm{prior}}-\sigma_{\theta ,j}^{\mathrm{prior}};\mu_{\theta ,j}^{\mathrm{prior}}+\sigma_{\theta ,j}^{\mathrm{prior}}]$ where $\mu_{\theta ,j}^{\mathrm{prior}}$ is the parameter expected value *a priori* (i.e. values reported in tables 7–9) and $\sigma_{\theta ,j}^{\mathrm{prior}}$ the marginal standard deviation *a priori* (as described in appendix C.4). We then sample the combined interval (i.e. $\theta \in \Pi_{\;j=1}^{\;j=N_{\theta }}I_{\theta ,j}$) using a Latin–hypercube sampling algorithm approximately 10^6^ times. We further use 20 conditioning intervals and we use the implementation of PAWN median sensitivity index available in SALib [66].

We calculate PAWN median sensitivity index associated with the oxygen concentration in the gel, the glucose concentration in the media, the VEGF concentration in the media and the cell density in the gel after 24 h. We obtain one sensitivity index per field that we then sum to obtain the global impact each parameter has on the model predictions that is used in figure 10.

## Appendix G. Bayesian experimental designs for vascular endothelial growth factor secretion

We devised four experimental designs, each one centred around varying one initial or boundary condition, i.e. the oxygen concentration at the air/media interface *C*_*a*/*m*_, the initial oxygen concentration *C*_0,ox_, the initial glucose concentration *C*_0,glu_ and initial cell density *C*_0,cell_ (table 12).

**Table 12.** Experimental designs tested.

parameter	units	design 1	design 2	design 3	design 4
*C*_*a*/*m*_	4.21 × 10^−4^ kg m^−3^	1, 3, 7	3	3	3
*C*_0,ox_	4.21 × 10^−4^ kg m^−3^	3	1, 3, 7	3	3
*C*_0,glu_	kg m^−3^	2	2	1, 2, 4	2
*C*_0,cell_	10^12^ cell m^−3^	30	30	30	15, 30, 60

Briefly, each design is composed of three points. All designs share one central point namely ($C_{a/m}=3\%O_{2}$, $C_{0,\mathrm{ox}}=3\%O_{2}$, *C*_0,glu_ = 2 kg m^−3^ and *C*_0,cell_ = 30 × 10^12^ cell m^−3^). Each design then adds two points, one located above and one located below the central point for each of the initial and boundary condition. For instance, the design centred around *C*_0,cell_ (Design 4 in table 12) has $C_{0,\mathrm{cell}}=30\times 10^{12}\;\mathrm{cell}\;m^{-3}$ as a central value for initial cell density and $C_{0,\mathrm{cell}}=60\times 10^{12}\;\mathrm{cell}\;m^{-3}$ and *C*_0,cell_ = 15 × 10^12^ cell m^−3^ as cell density values associated with points located above and below the central point, respectively.

The designs were chosen to reflect the one used for F7s and CTXs (table 9). Three points per design were chosen to mitigate computational costs. We note that in practice, any experimental design could be tested.

We then generate, for each experimental design, a synthetic experimental dataset. Here we do so by taking the posterior estimates reported in tables 5 and 6. We then solve equations (2.9)–(2.18) for the values of VEGF concentration in the media after 24 h for each VEGF secretion model reported in table 3. Similarly to the F7s dataset, we consider an additive noise with *σ*_*e*_ = 50 × 10^−9^ kg m^−3^.

Finally, we calculate the Kullback–Leiber divergence for each experimental design and each VEGF secretion model using the prior estimates reported in tables 8 and 7 and appendix C.4. The only difference with §2.3.6 is that the initial and boundary conditions are assumed to be known exactly to better highlight their impact. The results are then averaged over the VEGF secretion models and displayed in figure 13.
